# Effectiveness of electro‐acupuncture for cognitive improvement on Alzheimer's disease quantified via PET imaging of sphingosine‐1‐phosphate receptor 1

**DOI:** 10.1002/alz.14260

**Published:** 2024-09-25

**Authors:** Lu Wang, Lei Bi, Yifan Qiu, Guolong Huang, Peizhen Ye, Yongshan Liu, An Li, Xuan Yang, Peining Shen, Junfeng Wang, Qi Zeng, Hongyu Zhang, Shengqiao Li, Hongjun Jin

**Affiliations:** ^1^ Department of Chinese Medicine Oncology Cancer Center The Fifth Affiliated Hospital Sun Yat‐sen University Zhuhai China; ^2^ Guangdong Provincial Engineering Research Center of Molecular Imaging The Fifth Affiliated Hospital Sun Yat‐sen University Zhuhai China; ^3^ Guangdong‐Hong Kong‐Macao University Joint Laboratory of Interventional Medicine, The Fifth Affiliated Hospital Sun Yat‐sen University Zhuhai China; ^4^ Pharmaceutical Clinical Trails Office The Fifth Affiliated Hospital Sun Yat‐sen University Zhuhai China; ^5^ Department of Neurology The Fifth Affiliated Hospital Sun Yat‐sen University Zhuhai China

**Keywords:** Alzheimer's disease, astrocyte, electro‐acupuncture, microglia, micro‐positron emission tomography/computed tomography–magnetic resonance imaging, sphingosine‐1‐phosphate receptor 1

## Abstract

**INTRODUCTION:**

Electro‐acupuncture (EA) has demonstrated potential in improving mild‐to‐moderate dementia in clinics, but the underlying scientific target remains unclear.

**METHODS:**

EA was administered to *APP/PS1* Alzheimer's disease (AD) mice, with untreated AD, and wild type (WT) mice serving as controls. The efficacy of EA was assessed by the Morris water maze cognitive functional tests. Brain magnetic resonance imaging–positron emission tomography (PET) scans using [^18^F]TZ4877 targeting sphingosine‐1‐phosphate receptor 1 (S1PR1) and [^18^F]AV45 targeting amyloid beta fibrils were conducted. The correlation between regional brain PET quantifications and cognitive functions was analyzed.

**RESULTS:**

EA significantly improved cognitive and memory functions of AD (*p*  = 0.04) and reduced the uptake of [^18^F]TZ4877 in the cortex (*p*  = 0.02) and hippocampus (*p*  = 0.03). Immunofluorescence confirmed colocalizations of S1PR1 with glial fibrillary acidic protein and ionized calcium‐binding adaptor molecule‐1. Furthermore, immunohistochemistry showed a significant reduction of interleukin 1β and tumor necrosis factor α after EA treatment.

**DISCUSSION:**

EA may reverse AD by suppressing neuroinflammation, and the PET imaging of S1PR1 seemed potent in evaluating the treatment for AD patients

**Highlights:**

- Electro‐acupuncture (EA) was administered to APP/PS1 Alzheimer's disease (AD) mice, with untreated AD, and wild type (WT) mice serving as controls. The efficacy of EA was assessed by the Morris water maze cognitive functional tests and positron emission tomography (PET) imaging quantifications.
- PET tracer [^18^F]AV45 was used to detect amyloid beta deposition. An increased uptake of [^18^F]AV45 was found in AD compared to WT mice, with significance observed only in the cortex and not in the hippocampus. EA treatment exhibited a trend toward reduced [^18^F]AV45 uptake in AD mouse brains post‐treatment. However, statistical difference was not attained in most brain regions.
- EA “Baihui (DU20) and Sishencong (EX‐HN1)” significantly improved cognitive and memory functions of AD (*p* = 0.04). Brain magnetic resonance imaging p(MRI)–positron emission tomography (PET) quantifications revealed that significantly reduced the uptake of [^18^F]TZ4877 in the cortex (*p* = 0.02) and hippocampus (*p* = 0.03) after EA treatment.
- The correlation between PET quantifications and cognitive functions was analyzed and the most notable correlations were found between escape latency (reaction cognitive and memory behavior) and volume distribution (V_T_) quantifications of [^18^F]TZ4877. V_T_ quantifications of [^18^F]TZ4877 in key brain regions for cognitive and memory ability, such as the cortex and hippocampus, positively correlated with platform latency (cortex *p* < 0.01, *r* = 0.7102; hippocampus *p* < 0.01, *r* = 0.6891).
- Immunofluorescence confirmed colocalizations of S1PR1 with glial fibrillary acidic protein and ionized calcium‐binding adaptor molecule‐1 in the AD brain. And the EA treatment significantly reduced the signals in the cortex and hippocampus.
- Immunohistochemistry showed a significant reduction of interleukin 1β and tumor necrosis factor α after EA treatment. EA reversed AD by suppressing neuroinflammation in the cortex and hippocampus.
- The S1PR1 targeting PET tracer [^18^F]TZ4877 showed promise in evaluating the pathological progression of AD in clinical settings.

## 1 BACKGROUND

Alzheimer's disease (AD) represents a pressing global health challenge that is yet to be effectively addressed. Characterized by progressive cognitive dysfunction and behavioral impairment, AD poses a significant social and economic burden on health‐care systems worldwide. It is reported that ≈ 50 million individuals are currently suffering from AD, a number projected to triple by 2050.^1^, ^2^ Despite decades of research, significant advances in AD treatment remain elusive. Although recent antibody drugs, such as aducanumab and lecanemab, have shown promise in delaying the progression of AD, concerns persist regarding their long‐term efficacy and potential adverse effects.^3^ Therefore, there is a pressing need for a comprehensive treatment approach that encompasses not only pharmacological interventions but also alternative non‐drug therapies.^4^

Acupuncture, as a traditional Chinese medicine (TCM) therapy, involves the insertion of needles into specific acupoints to alleviate various diseases and has been practiced for thousands of years with beneficial interactions and few side effects.^5^ A 2002 report by the World Health Organization reviewed controlled clinical trials and confirmed the effectiveness of acupuncture in treating various diseases. In 2017, McDonald et al. reviewed the effectiveness of acupuncture in 122 treatments in 14 clinical fields and found that it was effective in 117 situations.^6^ The external structure of the body can reflect internal pathophysiological changes, by stimulating specific acupoints on the body through which it is possible to acquire therapeutic effects of certain symptoms. Although the precise mechanisms of meridians and acupoints are not fully understood, relevant research suggests that acupuncture can regulate body function through the nerve–endocrine–immune network.^7^, ^8^ Research has demonstrated the anti‐inflammatory effects of electro‐acupuncture (EA) in neurological disorders, such as brain injury, Parkinson's disease, dementia, post‐traumatic stress disorder, and so forth;^9^, ^10^ EA has also shown benefits in attenuating the progression of AD by regulating microglia‐ and astrocyte‐associated neuroinflammation.^11^, ^12^ However, further scientific evidence is needed to elucidate the underlying mechanisms and support EA as a viable alternative treatment strategy for AD.

Sphingosine‐1‐phosphate receptors (S1PRs) signaling plays a crucial role in the neuroimmune system. Among the S1P receptors, S1PR1 is the predominant receptor expressed in the brain, involved in inflammation, immunity, and brain function regulation.^13^, ^14^ In the central nervous system (CNS), S1PR1 is distributed in astrocytes, microglia, and oligodendrocytes,^15^ serving as a significant biomarker for neuroinflammation.^16^ Dysregulation of S1PR1 signaling pathways contributes to the progression of CNS inflammatory diseases,^17^ especially in AD. A previous study reported that S1PR1 expression is increased in the brains of patients with AD, and that inhibition of S1PR1 can alleviate AD‐related pathological progression.^18^ Positron emission tomography (PET) is a non‐invasive, dynamic, and sensitive method used to study pathophysiological neuroimmune systems in live subjects. In recent years, various imaging systems, including PET, have been used in acupuncture studies, which enables quantitative and imaging data collection and comparison at different time points before, after, and during acupuncture interventions. Such use enhances the design of high‐level acupuncture research schemes and facilitates the generation of high‐quality evidence.^19^ Currently, PET probes used in mechanistic research on acupuncture to improve cognitive impairment in AD include [^18^F]AV45 for evaluating amyloid fibers^20^ and [^18^F]FDG for measuring glucose metabolism,^21^ which may not fully meet the expectations for feasibly evaluating anti‐inflammation therapies for AD. Building upon our previous work with the S1PR1 targeting probe [^18^F]TZ4877, which was used to evaluate neuroinflammation in various physiological states and the effects of different therapeutic interventions,^22^, ^23^, ^24^ the present study aims to evaluate the effectiveness of EA therapy in *APP/PS1* transgenic mice as an AD model; it also seeks to elucidate the mechanisms underlying EA therapy's ability in delaying the progression of AD, particularly regarding its impact on neuroinflammation.

**RESEARCH IN CONTEXT**

1. **Systematic review**: Given the continued increasing demand for complementary treatment strategies to enhance cognitive functions for Alzheimer's disease (AD), acupuncture‐as a non‐pharmacologic therapy‐ holds considerable potential for its effectiveness, minimal side effects, and low cost for long‐term medical management. Electro‐acupuncture (EA) has shown promise in clinical treatment and animal studies of various neurological conditions, including AD. Unfortunately, scientific targeting of EA for neuroinflammation has remained explored.
2. **Interpretation**: The EA “Baihui (DU20, also DV20)‐Sishencong (EX‐HN1)” therapy significantly improved the cognitive and memory behavioral representations in the transgenic (APP/PS1) mouse model of AD, while reducing the expression of sphingosine‐1‐phosphate receptor 1 (S1PR1) in the cortex and hippocampus. Both [^18^F]TZ4877 (targeting S1PR1) and [^18^F]AV45 (targeting amyloid composition) showed increased uptake in the AD group; the increase of which can be reduced by EA treatment significantly only indicated by [^18^F]TZ4877 positron emission tomography (PET). There is a negative correlation between PET quantifications (S1PR1) in different brain regions and cognitive–memory behavior. Immunofluorescence results confirmed that S1PR1 colocalized with the astrocyte and microglial activations, which proved that S1PR1 is an important target associated with glial activations for AD. Targeting of the S1PR1 PET probe [^18^F]TZ4877 is more sensitive for evaluation of the effectiveness of AD treatments.
3. **Future directions**: The first application of the PET probe [^18^F]TZ4877 targeting S1PR1 demonstrated that EA “Baihui (DU20) and Sishencong (EX‐HN1)” therapy significantly improved the cognitive and memory behavior in a mouse model of AD by reducing the expression of S1PR1 in the cortex and hippocampus. From the perspective of non‐pharmacological alternative treatment, this proves that S1PR1 is an important neuroinflammation biomarker for evaluating the treatment effectiveness in AD. The study suggests that the probe [^18^F]TZ4877 targeting S1PR1 may be a more sensitive probe than [^18^F]AV45 with potential AD clinical application regarding prediction, efficacy evaluation, or prognostic evaluation.

## 2 METHODS

### 2.1 Animal grouping and intervention

**FIGURE 1:**
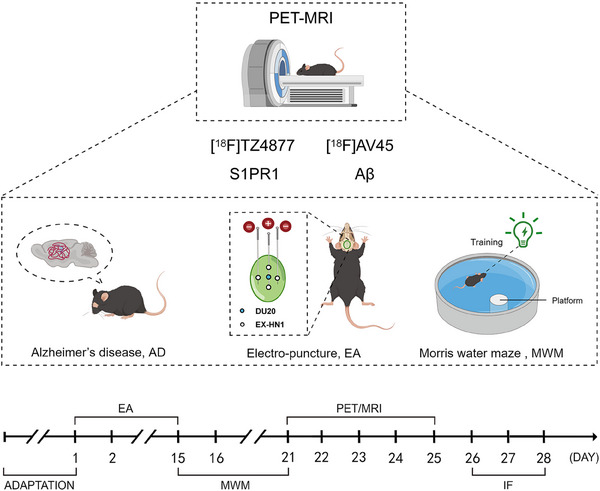
Diagrammatic representation of the experimental procedure (by Figdraw). Aβ, amyloid beta; EA, electro‐acupuncture; MRI, magnetic resonance imaging; PET, positron emission tomography; S1PR1, sphingosine‐1‐phosphate receptor 1.

Ten *APP/PS1* mice were randomly divided into the AD group and the EA group. Additionally, five age‐ and sex‐matched wild‐type (WT) *C57BL/6* mice were used as controls. Acupuncture was performed at five acupoints on the head of each mouse, including Baihui (GV20, also DU20, located at the midpoint between the auricular apices) and four Sishencong EX‐HN1 points (located at the four sides of DU20, ≈ 1.5 mm away), according to the Guidance of Experimental Acupuncture and Moxibustion^25^ (Figure 1; Table S1 in supporting information). Prior to acupuncture, animals were anesthetized with 1.5% isoflurane, and the area of operation was disinfected using 75% alcohol. Disposable sterile acupuncture needles were then inserted to a depth of 4 mm at an angle of 15° to 30°. The needle handles were connected to a HANSLH202 Electro‐acupuncture instrument (Table S1), and acupuncture was performed with the following parameters: continuous wave, frequency of 2 Hz, voltage of 2 V, and current intensity of 2 mA. Mice in the EA group received EA treatment for 20 minutes once daily for 15 days, while mice in the AD and WT group were subjected to the same anesthesia conditions without EA treatment.

### 2.2 Morris water maze experiment

The Morris water maze (MWM) experiment was based on a well‐established model, providing valuable insights into spatial learning and memory capabilities. The data were collected and analyzed using MWM system software and a Smart V3.0 camera system (Table S1).

#### 2.2.1 Visual platform experiment

The mice were trained for 1 day to eliminate visual interference and assess swimming ability. During the experiment, visual platforms were randomly placed in four quadrants, with two entry points on opposite sides of the platform quadrant. The swimming distance and time were recorded based on whether the animal was able to find the platform within 60 seconds and remained on it for 5 seconds. If the animal failed to find the platform within 60 seconds, it was then removed from the water and placed on the platform for 15 seconds to observe and remember the position, with an escape incubation period of 60 seconds.

#### 2.2.2 Hidden platform experiment

The mice underwent a hidden platform experiment for 4 days to assess changes in learning and memory. The hidden platform remained at a fixed position in a specific quadrant throughout the experiment. Subsequently, four fixed entry points were set, the daily training launching points were sorted randomly, and the distance between the entry point and the center of the sink was equal. The experimenter oriented the mouse with its head facing the tank wall and placed it in the water.

#### 2.2.3 Space exploration experiment

In this experiment, the platform was removed and mice were placed at the midpoint opposite to the targeted quadrant where it had been previously located, then each mouse was also observed for 60 seconds after entering the water, recording and analyzing the spatial memory ability.

### 2.3 Radiosynthesis of [^18^F]TZ4877 and [^18^F]AV45

The synthesis and quality control of [^18^F]TZ4877 were achieved by modifying a previous study.^24^ The nucleophilic reaction between the tosylate precursor and [^18^F]KF in acetonitrile with Kryptofifix 222 was followed by deprotection of the methoxymethyl group using hydrochloric acid (6 M). After purification using semi‐preparative high‐performance liquid chromatography (HPLC) combined with solid‐phase extraction, [^18^F]TZ4877 was formulated using 10% ethanol in saline with high radiochemical purity (> 95%), radiochemical yields (58.5 ± 12.7%), and specific activity (17.6 ± 5.3 GBq/µmol, *n* = 18, decay corrected to the end of synthesis; Figure S1 in supporting information). The synthesis and quality control of [^18^F]AV45 were produced from Guangdong Cyclotron Medical Science Co., Ltd. following a previous procedure^26^ and formulated using 10 mL ascorbic acid aqueous solution (0.15%) with high radiochemical purity (91.60%), radiochemical yields (62%), and specific activity (0.389 GBq/µmol, decay corrected to the end of synthesis; Table S2 in supporting information).

### 2.4 Magnetic resonance imaging acquisition

For magnetic resonance imaging (MRI) experiments, T2‐weighted brain images of mice were obtained using a DOTY 400 MHz 1H Rx surface coil on a 9.4 Tesla Bruker BioSpec small‐animal MRI system (Bruker BioSpin MRI, Ettlingen, Germany). Animals from each group were imaged individually. Initially, animals were anesthetized under 2% isoflurane in an induction chamber. The anesthetized mice were transferred to an MR‐compatible cradle and positioned in an MRI‐compatible head holder to minimize head motion. Anesthesia was then maintained at 1.5% isoflurane in the air throughout imaging. The respiration rate was monitored using a pressure pad placed under the animal's abdomen and the animal's body temperature was maintained by a warming pad (37°C) placed under the animal. The imaging was conducted on a horizontal bore 9.4 Biospec pre‐clinical MRI system (Bruker BioSpin MRI GmbH) equipped with shielded gradients (maximum gradient strength = 660 mT/m, rise time = 4750 T/m/s). An 86 mm quadrature volume resonator was used for transmission and a four‐element array cryocoil was used for signal reception (Cryoprobe, Bruker, BioSpin).

### 2.5 PET imaging

PET/computed tomography (CT) imaging studies were performed on the WT (*n* = 5), AD (*n* = 5), and the EA group (*n* = 5) for [^18^F]TZ4877 and [^18^F]AV45 separately. The interval between [^18^F]TZ4877 and [^18^F]AV45 imaging was 3 days. The mice were anesthetized with 1.5% to 2% isoflurane inhalation and intravenously injected with 150 µL of 8.2 ± 1.69 MBq/mouse [^18^F]TZ4877 or 8.7 ± 1.47 MBq/mouse [^18^F]AV45. A dynamic 0‐ to 30‐minute micro‐PET/CT (Mediso nanoScan PET/CT imaging system, Mediso Inc.) scan protocol was performed immediately after [^18^F]TZ4877 or [^18^F]AV45 injection through vein. Dynamic PET images were reconstructed into 27 frames (10 × 3 seconds, 3 × 10 seconds, 4 × 60 seconds, and 10 × 150 seconds). PET images (voxel size = 0.4 mm) were reconstructed using ordered subset expectation maximization (OSEM) algorithm with CT attenuation correction and isotope decay correction.

### 2.6 PET data analysis

Through the co‐registration of PET and CT, PET data of original [^18^F]TZ4877 and [^18^F]AV45 were quantified using a PMOD Biomedical Image Quantification System (Table S1). The PMOD Fusion Tool was used to obtain the blood input by drawing the region of interest (ROI) of the left ventricle. The volume of interest (VOI) consisting of at least three ROIs on the target area was obtained. The Mouse Brain Atlas of Ma–Benveniste–Mirrione in the PNROD from PMOD was used for mouse brain segmentation. PET data in target region was expressed as standardized uptake value (SUV). SUV was calculated as the activity concentration within the VOI (Bq/mL) divided by injected activity (Bq) and multiplied by body weight (g). The VOI was plotted on each brain region and the data at 30 minutes post‐injection for static PET/CT to obtain SUV values, and over 30 minutes for dynamic PET/CT to obtain the time activity curve (TAC). The TAC curves of different brain regions were recorded and used to calculate volume distribution (*V*_T_) through the Logan plot (LoganAIF).^27^ TACs of the left ventricle were obtained from the original micro‐PET data and used for arterial input function. The goodness of fit was evaluated with r^2^.

### 2.7 Immunofluorescence staining and immunohistochemistry

Immunofluorescence (IF) staining for S1PR1 and glial fibrillary acidic proteins (GFAP) or ionized calcium‐binding adaptor molecule‐1 (IBA‐1) was conducted on brain tissue collected post‐PET experiments. Mice were euthanized and their brains were perfused with 4% paraformaldehyde and phosphate‐buffered saline (PBS). Brain tissue was removed to place in a cassette, with optimal cutting temperature compound frozen in liquid nitrogen, and then stored at –80°C. Brain tissue was sliced into 6 µm sections by using a frozen microtome and then permeabilized with 0.3% triton‐100 for 30 minutes. After washing with PBS, the slices were blocked with 10% bovine serum albumin and incubated overnight at 4°C with S1PR1, GFAP, and IBA‐1 primary antibodies (Table S2). After three washes with PBS, the tissues were incubated with a mixture of secondary antibodies against S1PR1, GFAP, IBA‐1, and DAPI at room temperature for two hours, and coverslips were added. Also, immunohistochemistry for interleukin (IL)‐1β and tumor necrosis factor alpha (TNF‐α) was conducted on the paraffin sections of brain tissue. All the above reagent details are described in Table S2. Images were acquired using a ZEISS LSM 880 laser scanning confocal microscope system (Table S1).

### 2.8 Statistical analysis

Statistical analyses were performed using GraphPad Prism 9.0 (GraphPad Software, Inc.) and SPSS 26.0 (IBM). Data are expressed as mean and standard deviation (± SD). For comparison between multiple groups, one‐way analysis of variance (ANOVA) was used for normal distribution. After analysis of homogeneity of variance, the least significant difference (LSD) test was used for pairwise comparison between groups, and the rest were compared by non‐parametric test. Differences were considered statistically significant at *p* < 0.05.

## 3 RESULTS

### 3.1 EA treatment reduced the escape latency of AD mice

**FIGURE 2:**
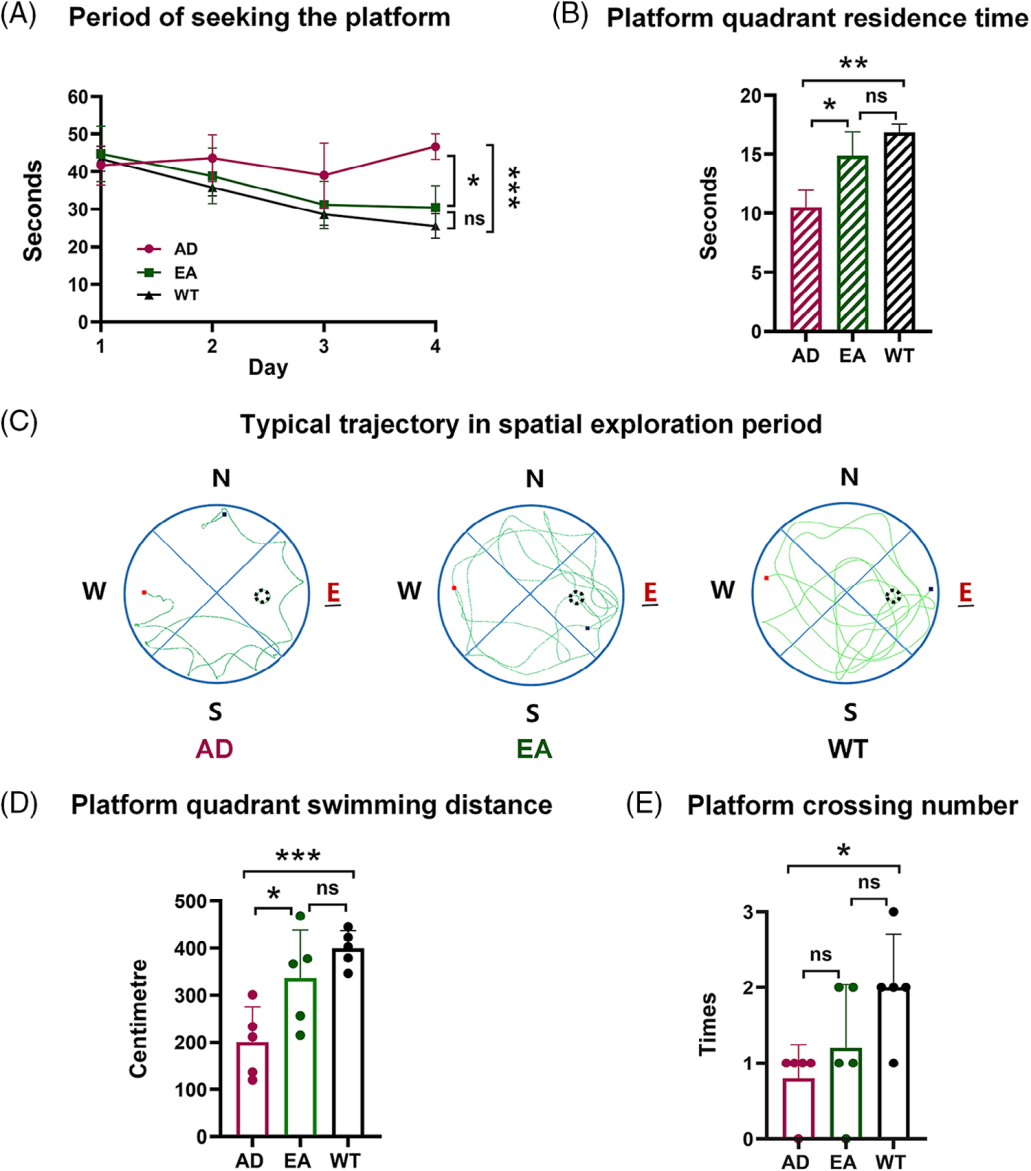
EA treatment improved the cognitive learning and memory ability of AD mice. A, The trend of escape latency of mice in the hidden platform experiment. B, The exploration time of mice in the platform quadrant experiment. C, The typical trajectory of three groups in a spatial exploration experiment. D, The exploration distance in the platform quadrant of each group in the space exploration trial (WT vs. AD, *p* < 0.01; EA vs. AD, *p* < 0.05; WT vs. EA, *p* > 0.05). E, The platform crossing numbers of each group in the space exploration trial (WT vs. AD, *p* < 0.05; EA vs. AD and WT vs. EA, *p* > 0.05; *n* = 5). *p < 0.05, **p < 0.01, ***p < 0.001, ns: *p* > 0.05. AD, Alzheimer's disease; EA, electro‐acupuncture; WT, wild type.

In the hidden platform experiment, the escape latency of mice in each group showed a decreasing trend over the training days. Compared to the WT group, the escape latency of mice in the AD group was significantly prolonged (46.63 ± 6.34 seconds in AD vs. 25.54 ± 6.50 seconds in WT, *n* = 5, *p* = 0.002), confirming that the 8‐month‐old *APP/PS1* mice (genotyped in supplementary materials Figure S2 in supporting information) had obvious impairment in learning and memory. Surprisingly, the escape latency in EA‐treated mice (30.42 ± 10.58 seconds in EA vs. 46.63 ± 6.34 seconds in AD, *n* = 5, *p* = 0.04) was significantly shorter than that of the untreated AD group; and did not significantly differ from the WT group (30.42 ± 10.58 seconds in EA vs. 25.54 ± 6.50 seconds in WT, *n* = 5, *p* = 0.44), further suggesting that the EA treatment was able to reverse the learning and memory ability of AD mice to close to the WT mice (Figure 2A).

### 3.2 EA treatment improved the spatial exploration time of AD mice

Similarly, a spatial exploration experiment was performed after the hidden platform experiment to test the spatial memory of the mice after 4 days of training. The longer the swimming time in the platform quadrant, the better the memory retention. In this experiment, the exploration time was significantly (*p* = 0.04) shorter in the AD group (10.51 ± 2.52 seconds, *n* = 5) than in the WT group (16.85 ± 1.47 seconds, *n* = 5). However, the spatial exploration time was significantly (*p* = 0.02) longer in the EA group (14.88 ± 4.03 seconds, *n* = 5) compared to the AD group (10.51 ± 2.52 seconds). Despite the longest exploration time in the WT group (16.85 ± 1.47 seconds), there was no significant (*p* = 0.11) difference compared to the EA group (14.88 ± 4.03 seconds; Figure 2B). The map of a typical mouse tour track showed the spatial memory ability of mice in different groups (WT > EA > AD, Figure 2C).

Swimming distance in the platform quadrant also served as an indicator of spatial cognitive and memory abilities. The longer the swimming distance in the platform, the better the memory retention. The longest swimming distance was in the WT group (40.50 ± 4.28 cm), followed by the EA group (33.70 ± 9.07 cm), and the shortest was in the AD group (20.07 ± 6.62 cm; WT vs. AD: *p =* 0.0008; EA vs. AD: *p* = 0.04; WT vs. EA: *p* = 0.21, *n* = 5) as shown in Figure 2D. Similarly, the greater the number of platform crossings, the better the memory retention (2 ± 0.63 times in the WT group, 1.2 ± 0.75 times in the EA group, and 0.8 ± 0.4 times in the AD group, WT vs. AD: *p =* 0.01; EA vs. AD: *p* = 0.37; WT vs. EA: *p* = 0.14, *n* = 5, respectively; Figure 2E).

### 3.3 EA treatment reduced uptake of [^18^F]TZ4877 in the cortex and hippocampus of AD mice

**FIGURE 3:**
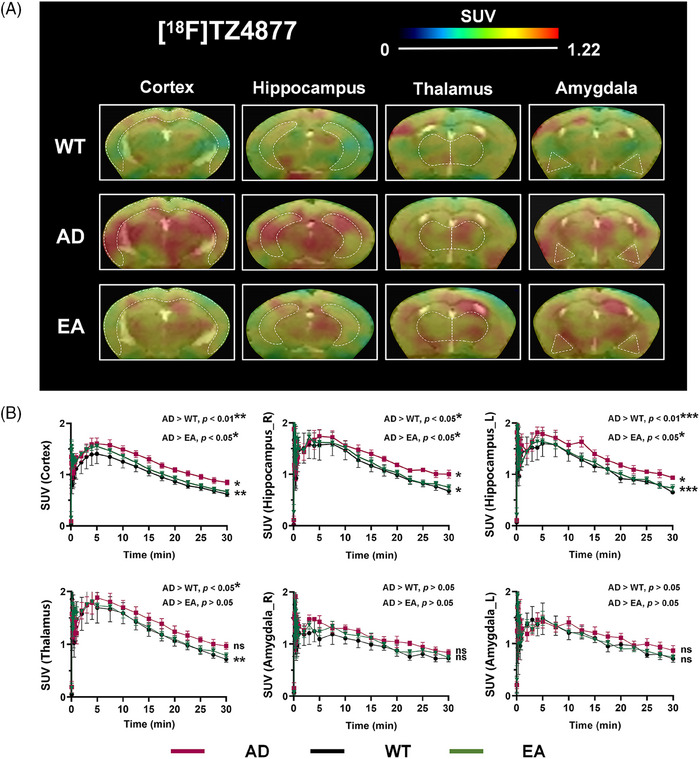
The PET‐MRI imaging and uptake comparisons of [^18^F]TZ4877 among WT, AD, and EA mice. A, The representative PET‐MRI images of [^18^F]TZ4877 in the brain regions of WT, AD, and EA mice. B, The averaged TACs of [^18^F]TZ4877 in the brain regions from the WT (black lines), AD (red lines), and EA (green lines) groups, *n* = 5. *p < 0.05, **p < 0.01, ***p < 0.001, ns: *p* > 0.05. AD, Alzheimer's disease; EA, electro‐acupuncture; MRI, magnetic resonance imaging; PET, positron emission tomography; SUV, standardized uptake values; TACs, time activity curves; WT, wild type.

To assess the potential impact of EA treatment on neuroinflammation in these mice in vivo, PET/CT/MRI brain imaging and quantifications were performed. As shown in Figure 3 and Figure S3 in supporting information, compared to the WT group (*n* = 5), a notable increase of [^18^F]TZ4877 uptake was found in AD mice (*n* = 5) at 30 minutes post‐injection (Table 1). Furthermore, the increased uptake in the AD brain can be visibly reduced especially in the cortex and hippocampus by EA (*n* = 5) treatment as illustrated in Figure 3A, and the quantification of SUV is presented in Table 1. Although there was a slightly higher SUV in EA mice than in WT mice, the disparity was not statistically significant.

**TABLE 1:** The SUV comparisons of [^18^F]TZ4877 (S1PR1: AD, *n* = 5; EA, *n* = 5; WT, *n* = 5) and [^18^F]AV45 (Aβ: AD, *n* = 5; EA, *n* = 5; WT, *n* = 3) in AD, WT, and EA groups at 30 minutes post‐injection.

Brain areas	AD	EA	WT	AD vs. WT *p* value	AD vs. EA *p* value	EA vs. WT *p* value
S1PR1 cortex	0.85 ± 0.10	0.67 ± 0.08	0.62 ± 0.09	<0.01**	0.02*	0.43
S1PR1 hippocampus_R	1.00 ± 0.16	0.75 ± 0.11	0.67 ± 0.12	0.01*	0.03*	0.41
S1PR1 hippocampus_L	0.94 ± 0.07	0.73 ± 0.14	0.65 ± 0.07	<0.01***	0.03*	0.33
S1PR1 atriatum	0.94 ± 0.08	0.77 ± 0.10	0.72 ± 0.12	0.02*	0.03*	0.61
S1PR1 thalamus	0.97 ± 0.13	0.78 ± 0.11	0.72 ± 0.11	0.02*	0.07	0.40
S1PR1 cerebellum	0.92 ± 0.11	0.74 ± 0.11	0.71 ± 0.13	0.04*	0.05	0.71
S1PR1 basal forebrain	0.83 ± 0.09	0.78 ± 0.10	0.71 ± 0.14	0.18	0.53	0.40
S1PR1 hypothalamus	0.86 ± 0.06	0.72 ± 0.09	0.72 ± 0.18	0.17	0.03*	0.95
S1PR1 amygdala_R	0.84 ± 0.12	0.74 ± 0.10	0.73 ± 0.14	0.25	0.25	0.85
S1PR1 amygdala_L	0.87 ± 0.19	0.74 ± 0.09	0.71 ± 0.13	0.21	0.27	0.69
S1PR1 brain stem	0.94 ± 0.12	0.83 ± 0.05	0.79 ± 0.14	0.15	0.12	0.65
S1PR1 central_gray	1.02 ± 0.13	0.82 ± 0.12	0.67 ± 0.06	<0.01***	0.05	0.05
S1PR1 midbrain	1.03 ± 0.12	0.83 ± 0.15	0.78 ± 0.13	0.02*	0.07	0.60
Aβ cortex	0.89 ± 0.10	0.78 ±0 .04	0.66 ± 0.10	0.04*	0.07	0.08
Aβ hippocampus_R	1.90 ± 0.07	0.90 ± 0.04	0.73 ± 0.14	0.07	0.51	0.03*
Aβ hippocampus_L	0.94 ± 0.11	0.90 ± 0.07	0.71 ± 0.11	0.09	0.49	0.11
Aβ striatum	0.92 ± 0.12	0.84 ± 0.05	0.68 ± 0.11	0.04*	0.26	0.04*
Aβ thalamus	0.92 ± 0.14	0.86 ± 0.07	0.72 ± 0.06	0.08	0.45	0.04*
Aβ cerebellum	0.96 ± 0.11	0.88 ± 0.04	0.71 ± 0.10	0.03*	0.24	0.02*
Aβ basal forebrain	0.89 ± 0.13	0.83 ± 0.03	0.69 ± 0.14	0.12	0.37	0.12
Aβ hypothalamus	0.93 ± 0.14	0.81 ± 0.03	0.69 ± 0.11	0.07	0.13	0.09
Aβ amygdala_R	0.88 ± 0.16	0.86 ± 0.06	0.68 ± 0.12	0.16	0.81	0.05
Aβ amygdala_L	0.93 ± 0.11	0.81 ± 0.04	0.75 ± 0.18	0.17	0.07	0.56
Aβ brain stem	1.01 ± 0.10	0.91 ± 0.05	0.74 ± 0.11	0.02*	0.12	0.04*
Aβ central_gray	0.95 ± 0.17	0.92 ± 0.11	0.65 ± 0.08	0.04*	0.77	0.02*
Aβ midbrain	0.98 ± 0.11	0.90 ± 0.06	0.71 ± 0.10	0.02*	0.19	0.03*

Abbreviations: Aβ, amyloid beta; AD, Alzheimer's disease; EA, electro‐acupuncture; S1PR1, sphingosine‐1‐phosphate receptor 1; SUV, standardized uptake value; *V*_T_, volume distribution; WT, wild type.

* *p* < 0.05.

** *p* < 0.01.

*** *p* < 0.001.

### 3.4 EA treatment declines amyloid beta expression in brain regions of AD mice

**FIGURE 4:**
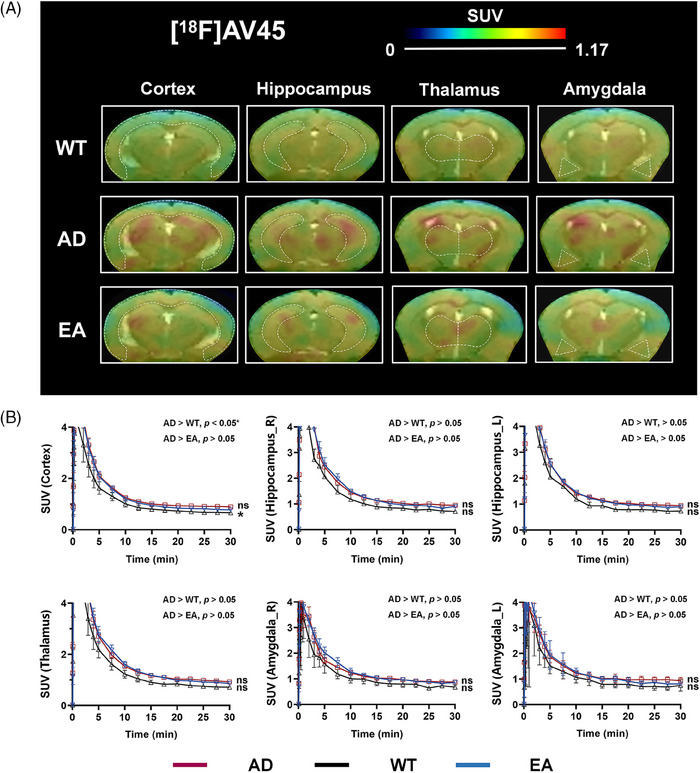
The PET‐MRI imaging and uptake comparisons of [^18^F]AV45 among WT, AD, and EA mice. A, The representative PET images showed the uptake of [^18^F]AV45 in brain regions of WT, AD, and EA mice. The images of different brain regions are indicated by white dotted lines. B, TAC shows the uptake of [^18^F]AV45 in WT (black lines), AD (red lines), and EA (blue lines) mice. *n* = 3 in WT, and *n* = 5 in AD and EA, respectively. *p < 0.05, **p < 0.01, ***p < 0.001, ns: *p* > 0.05. AD, Alzheimer's disease; EA, electro‐acupuncture; MRI, magnetic resonance imaging; PET, positron emission tomography; SUV, standardized uptake values; TAC, time activity curve; WT, wild type.

To further investigate the efficacy of EA in AD, PET tracer [^18^F]AV45 was used to detect amyloid beta (Aβ) deposition in mouse brains. As shown in Figure 4 and Figure S4 in supporting information, an increased uptake of [^18^F]AV45 was found in AD mice (*n* = 5) compared to WT mice (*n* = 3), with significance observed only in the cortex and not in the hippocampus. Similarly, EA treatment (*n* = 5) exhibited a trend toward reduced [^18^F]AV45 uptake in AD mouse brains post‐treatment. However, statistical difference was not attained in most brain regions (Table 1).

### 3.5 The *V*_T_ quantifications

SUV quantification based on the 30 minutes post‐injection may not precisely reflect the tracer binding kinetics; *V*_T_ provides a more comprehensive assessment. *V*_T_ was calculated to compare the binding of [^18^F]TZ4877 or [^18^F]AV45 in regions of the brain among the EA, AD group, and WT groups. For [^18^F]TZ4877, significant increases in *V*_T_ were observed in AD (*V*_T_^cortex^ 1.59 ± 0.25, *V*_T_^Hippocampus_R^ 1.00 ± 0.20; *V*_T_^Hippocampus_L^ 0.97 ± 0.21) over WT mice (*V*_T_^cortex^ 0.95 ± 0.25, *V*_T_^Hippocampus_R^ 1.81 ± 0.37; *V*_T_^Hippocampus_L^ 1.72 ± 0.35). Also, there was a statistically decreased *V*_T_ in EA (*V*_T_^cortex^ 1.16 ± 0.20, *V*_T_^Hippocampus_R^ 1.25 ± 0.23; *V*_T_^Hippocampus_L^ 1.24 ± 0.25) compared to AD mice, while the *V*_T_ analysis of [^18^F]AV45 did not show any significant reductions (Table 2). As shown in Figure S5 in supporting information, the degree of separation of oblique lines with Logan plots in the cortex and hippocampus of EA (*n* = 5) or AD mice (*n* = 5) showed more pronounced changes in [^18^F]TZ4877 binding. Overall, these data suggested that the binding of these two tracers was reduced in EA versus AD mice; significant statistical reductions were observed only in the binding of [^18^F]TZ4877, especially in the cortex and hippocampus, whereas the binding of [^18^F]AV45 was not.

**TABLE 2:** The *V*_T_ comparisons of [^18^F]TZ4877 (S1PR1: AD, *n* = 5; EA, *n* = 5; WT, *n* = 5) and [^18^F]AV45 (Aβ: AD, *n* = 5; EA, *n* = 5; WT, *n* = 3) in AD, WT, and EA groups at 30 minutes post‐injection.

Brain areas	AD	EA	WT	AD vs. WT p value	AD vs. EA *p* value	EA vs. WT *p* value
S1PR1 cortex	1.59 ± 0.25	1.16 ± 0.20	0.95 ± 0.25	<0.01**	0.03*	0.23
S1PR1 hippocampus_R	1.81 ± 0.37	1.25 ± 0.23	1.00 ± 0.20	<0.01**	0.04*	0.14
S1PR1 hippocampus_L	1.72 ± 0.35	1.24 ± 0.25	0.97 ± 0.21	<0.01**	0.05	0.13
S1PR1 striatum	1.69 ± 0.37	1.29 ± 0.23	0.98 ± 0.25	0.01*	0.11	0.10
S1PR1 thalamus	1.82 ± 0.44	1.37 ± 0.26	1.11 ± 0.26	0.02*	0.12	0.19
S1PR1 cerebellum	1.74 ± 0.35	1.28 ± 0.23	1.05 ± 0.26	0.01*	0.06	0.23
S1PR1 basal forebrain	1.57 ± 0.42	1.23 ± 0.28	0.95 ± 0.25	<0.01**	0.09	0.14
S1PR1 hypothalamus	1.61 ± 0.35	1.19 ± 0.21	1.05 ± 0.31	0.04*	0.08	0.48
S1PR1 amygdala_R	1.50 ± 0.34	1.17 ± 0.29	0.86 ± 0.22	0.01*	0.19	0.13
S1PR1 amygdala_L	1.58 ± 0.34	1.24 ± 0.24	0.98 ± 0.19	0.02*	0.14	0.14
S1PR1 brain stem	1.80 ± 0.33	1.41 ± 0.20	1.17 ± 0.27	0.02*	0.07	0.19
S1PR1 central_gray	1.81 ± 0.48	1.32 ± 0.24	1.05 ± 0.25	0.02*	0.11	0.16
S1PR1 midbrain	1.84 ± 0.49	1.35 ± 0.25	1.10 ± 0.22	0.02*	0.11	0.17
Aβ cortex	1.99 ± 0.48	1.81 ± 0.34	1.33 ± 0.29	0.11	0.55	0.13
Aβ hippocampus_R	2.14 ± 0.57	1.99 ± 0.40	1.44 ± 0.33	0.14	0.68	0.13
Aβ hippocampus_L	2.11 ± 0.52	2.05 ± 0.44	1.43 ± 0.31	0.13	0.87	0.11
Aβ striatum	2.06 ± 0.52	1.96 ± 0.36	1.39 ± 0.30	0.13	0.74	0.10
Aβ thalamus	2.20 ± 0.61	2.12 ± 0.41	1.48 ± 0.24	0.14	0.83	0.08
Aβ cerebellum	2.11 ± 0.53	2.04 ± 0.41	1.45 ± 0.27	0.14	0.85	0.10
Aβ basal forebrain	1.95 ± 0.49	1.91 ± 0.38	1.34 ± 0.29	0.14	0.90	0.10
Aβ hypothalamus	2.09 ± 0.53	1.92 ± 0.42	1.39 ± 0.33	0.13	0.63	0.16
Aβ amygdala_R	1.83 ± 0.36	1.81 ± 0.36	1.28 ± 0.24	0.09	0.92	0.10
Aβ amygdala_L	1.95 ± 0.47	1.76 ± 0.36	1.31 ± 0.32	0.12	0.54	0.17
Aβ brain stem	2.23 ± 0.59	2.10 ± 0.42	1.51 ± 0.35	0.15	0.73	0.13
Aβ central_gray	2.22 ± 0.54	2.21 ± 0.52	1.55 ± 0.26	0.14	0.99	0.12
Aβ midbrain	2.20 ± 0.59	2.12 ± 0.40	1.49 ± 0.28	0.14	0.82	0.08

Abbreviations: Aβ, amyloid beta; AD, Alzheimer's disease; EA, electro‐acupuncture; S1PR1, sphingosine‐1‐phosphate receptor 1; *V*_T_, volume distribution; WT, wild type.

* *p* < 0.05.

** *p* < 0.01.

### 3.6 Positive correlation between cognitive–memory behavior and *V*_T_ quantifications of [^18^F]TZ4877

**FIGURE 5:**
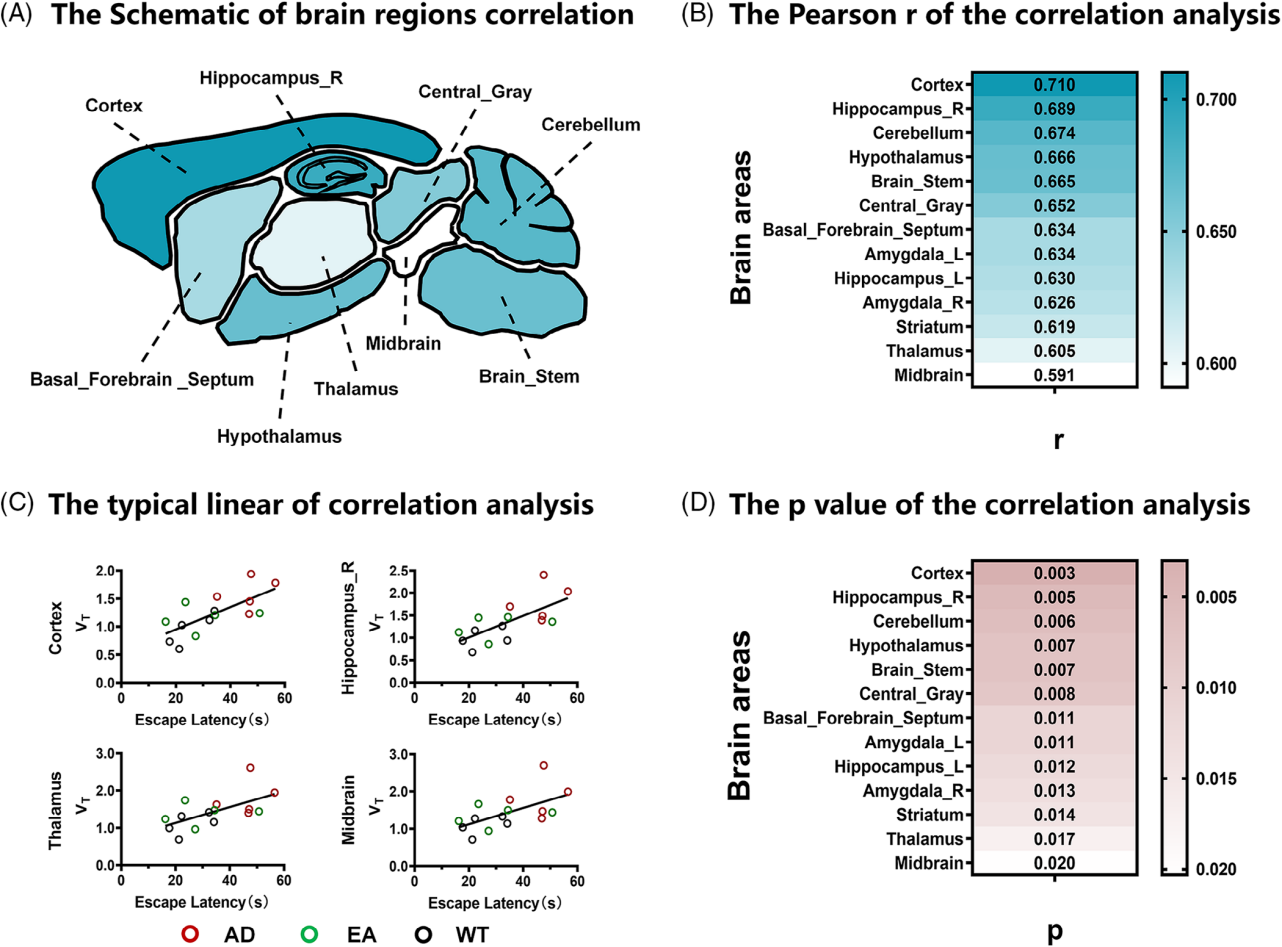
The correlations between cognitive–memory behavior and *V*_T_ quantifications of [^18^F]TZ4877. A, The schematic representation in the correlation of different brain regions. B, The *p*‐value in the correlation of *V*_T_ ([^18^F]TZ4877) in different brain areas and escape latency in the hidden platform experiment. C, The positive linear correlation between *V*_T_ ([^18^F]TZ4877) and escape latency. D, The Pearson *r* in the correlation of *V*_T_ ([^18^F]TZ4877) in different brain regions and escape latency in the hidden platform experiment. AD, Alzheimer's disease; EA, electro‐acupuncture; *V*_T_, volume distribution; WT, wild type.

To further clarify the association between the neuroinflammatory target S1PR1 and cognitive–memory behavior across various brain regions by examining the correlation between the cognitive–memory behavior and *V*_T_ quantifications of [^18^F]TZ4877 was investigated. We explored all behavioral correlations and found the most notable correlations between escape latency (reaction cognitive and memory behavior) and *V*_T_ quantifications of [^18^F]TZ4877. The results (Figure 5 and Figure S6 in supporting information) showed the *V*_T_ quantifications of [^18^F]TZ4877 in key brain regions for cognitive and memory ability, such as the cortex and hippocampus positively correlating with platform latency (cortex *p* < 0.01, *r* = 0.7102; hippocampus *p* < 0.01, *r* = 0.6891), indicating that higher *V*_T_ values were associated with longer periods to find the platform.

### 3.7 The IF staining showed a colocalization of S1PR1 with neuroinflammatory markers

**FIGURE 6:**
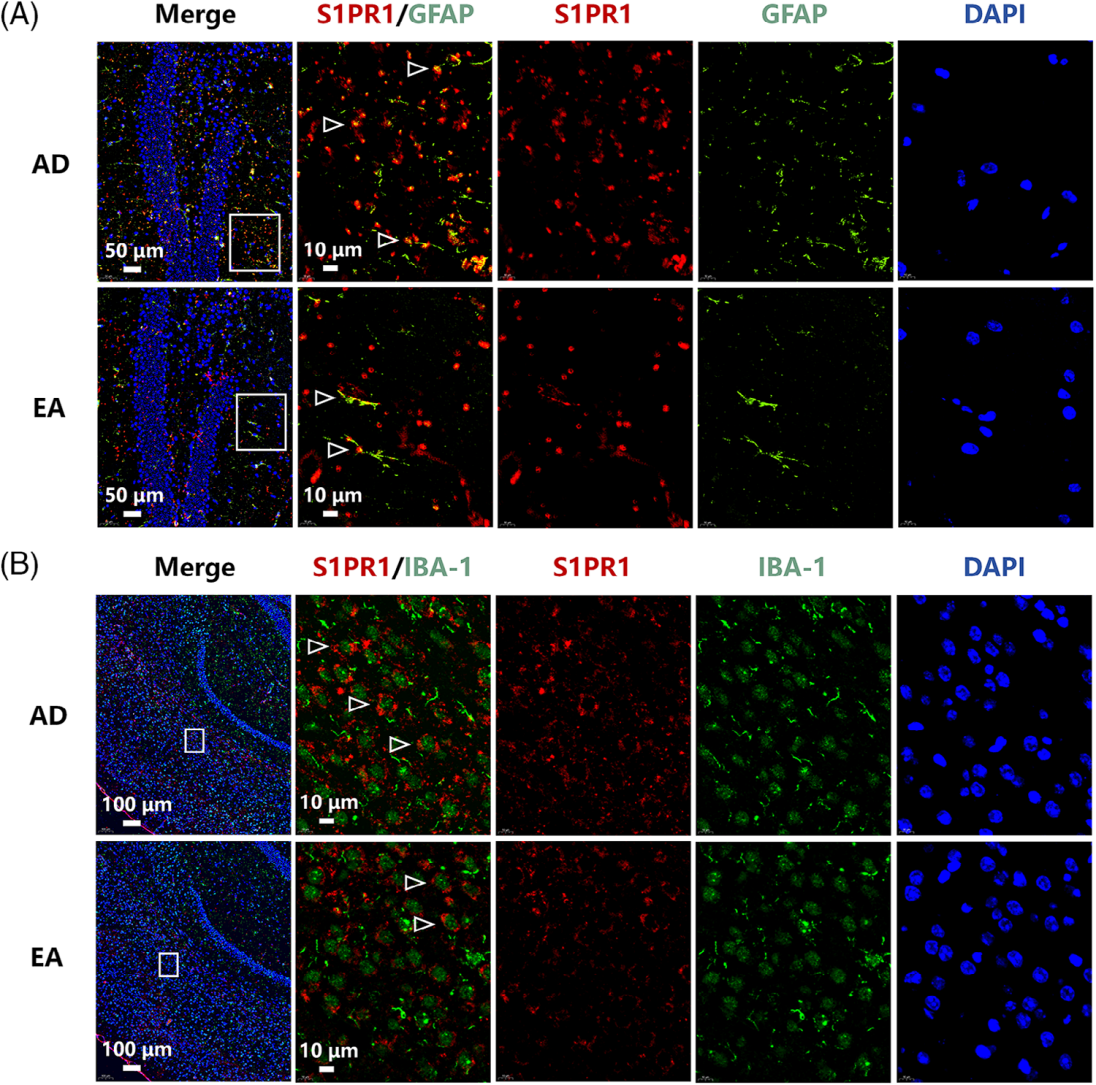
Colocalization of S1PR1 with GFAP and IBA‐1. A, Multiple IF staining showed colocalizations (white arrows) of S1PR1 (red), GFAP (green for astrocytes), and DAPI (blue) in hippocampus of AD and EA mice; (B) multiple IF staining showed colocalization (white arrows) of S1PR1 (red), IBA‐1 (green for microglia), and DAPI (blue) in cortex of AD and EA mice. The EA‐treated mice showed a significant reduction of the colocalization (white arrows) (A,B). AD, Alzheimer's disease; EA, electro‐acupuncture; GFAP, glial fibrillary acidic protein; IBA‐1, ionized calcium‐binding adaptor molecule‐1; IF, immunofluorescence; S1PR1, sphingosine‐1‐phosphate receptor 1.

The post‐PET immunofluorescence staining confirmed S1PR1 expression and explored its relationship with neuroinflammation markers. Cortex and hippocampus tissues of AD mice showed colocalization of S1PR1 (red) with neuroinflammation marker GFAP for astrocytes and IBA‐1 for microglia (Figure 6 and Figure S7 in supporting information). Colocalization in EA mice was significantly reduced (Figure 6A,B), suggesting suppression of astrocytes or microglia activation, which thereby can be assessed by PET imaging via [^18^F]TZ4877 targeting to S1PR1 receptor.

### 3.8 The immunohistochemistry showed a reduction of IL‐1β and TNF‐α after EA treatment

The post‐PET immunohistochemistry confirmed a significant reduction of IL‐1β and TNF‐α in the AD brain, especially in the cortex and hippocampus after EA treatment (Figure S8 in supporting information). Paraffin sections of brain cortex and hippocampus tissue from AD mice showed expression of IL‐1β (Figure S8A,B) and TNF‐α (Figure S8C,D). However, in EA‐treated mice, expression was significantly reduced (Figure S8A–D), suggesting EA's suppression of inflammation in AD brain tissue.

## 4 DISCUSSION

Neuroinflammation is an important factor in accelerating the pathological process of AD. As a neuroinflammation mediator, S1PR1 has been reported to be involved in the development of AD, although direct evidence from on‐site imaging is lacking. In this study, the [^18^F]TZ4877 probe targeting S1PR1 and [^18^F]AV45 probe targeting Aβ were used to evaluate the mechanisms by which EA therapy improves AD. On‐site imaging (Figures 3 and 4) revealed increased tracer uptake both for [^18^F]TZ4877 and [^18^F]AV45 in AD mouse brain regions compared to WT, while [^18^F]TZ4877 was more pronounced. After EA treatment, uptakes of [^18^F]TZ4877 and [^18^F]AV45 were both reduced with concomitant improvement in AD symptoms (Tables 1 and 2). Similarly, the reduction in [^18^F]TZ4877 was also more notable, which may suggest that the [^18^F]TZ4877 may be a more potentially sensitive probe in evaluating the pathological progression of AD.

Glial cells, including microglia and astrocytes, significantly influence impact on the pathology of AD. Previous knowledge^28^ has generally attributed CNS immunosurveillance only to resident microglia as resident macrophages of the CNS; they are involved in the process of maintaining brain homeostasis. The concept of microglia is crucial in the early stage of the disease research and can even be used as a potential therapeutic target. Astrocytes have been identified as key cortical regulators in the arousal state^29^ and are considered indispensable players in cognitive function.^30^ Our IF results (Figure 6 and S7) indicated the co‐localization of S1PR1 with GFAP (astrocyte) or IBA‐1 (microglia), further suggesting EA may improve cognitive and memory functions of AD by modulating astrocyte or microglia‐related functions. Additionally, the results of immunohistochemistry for IL‐1β and TNF‐α (Figure S8) suggest that EA may alleviate AD pathology by suppressing astrocyte or microglia‐related inflammation. To further explore the association between S1PR1 and cognitive–memory behavior, correlation analysis was conducted between *V*_T_ quantifications across various brain regions of [^18^F]TZ4877 and the escape latency (Figure S6). The result revealed that, as a widespread neuroinflammatory target, S1PR1 in brain areas was positively associated with AD cognitive and memory behavior impairment; the higher the S1PR1 expressed the longer the platform latency period (the time taken to find the platform), especially in the cortex and hippocampus. Additionally, a stronger correlation was observed in the right hippocampal than in the left, possibly due to its greater contribution to spatial memory.^31^, ^32^ EA treatment also mainly functions by decreasing S1PR1 expression in the cortex and hippocampus (a more pronounced effect observed on the right hippocampus), which provides evidence for EA's efficacy in improving cognitive and memory behavior in AD mice by acting on S1PR1 in the cortex and hippocampus (Tables 1 and 2).

The demand for complementary alternative medicine has increased significantly in recent decades, particularly for chronic and incurable diseases. According to TCM, the overall human body may encounter pathophysiological attacks and systemically fight back as a whole, making it an integrated medicine for living subjects. Patients are increasingly seeking therapies that are both more effective and with fewer side effects, offering new hope in managing their diseases. Current studies of acupuncture treatment for AD commonly use acupoints include Baihui (GV20), Yingtang (EX‐HN3), Shenshu (BL23), ZuSanli (ST36), and Shuigou (GV26).^33^ Given the focus on the brain in our study, we selected acupoints based on traditional meridian mapping on the brain, for example, Baihui (GV20, also DU20) and Sishencong (EX‐HN1), and also based on clinical evidence in managing mild to moderate AD using these two acupoints^34^ to better accommodate the use of the penetrating needling method combined with EA, representing an innovative improvement. To illustrate EA's mechanism in AD, the PET probe [^18^F]TZ4877 was used to assess S1PR1 expression in AD mice with or without EA treatment. Surprisingly, there was a significant decrease in [^18^F]TZ4877 levels in AD mice treated with EA, providing compelling evidence for EA's effectiveness in AD therapy (Figure 3). It is challenging to study TCM because its clinical practices defy the dominant biomedical paradigm. However, such challenges are pivotal in fostering continuous scientific innovation and breakthroughs. It is imperative to identify useful biomarkers for discerning the varying efficacies of acupuncture treatments. Liu et al.^8^ demonstrated that PROKR2^Cre‐^marked sensory neurons are the neuroanatomical basis of EA. In this study, we focused on S1PR1, an important biomarker for neuroinflammation. Our findings suggest that S1PR1 holds promise as a biomarker for evaluating acupuncture efficacy in diseases. The study's innovation lies in its pioneering use of S1PR1 PET probe [^18^F]TZ4877 to evaluate the efficacy of EA treatment in AD, underscoring the potential clinical utility of targeting S1PR1 PET in the diagnosis and treatment of AD.

There are still limits to this study, for instance, the sample size of AD animals is relatively small. However, despite this constraint, the identification of statistical discrepancies in *V*_T_ of PET tracer [^18^F]TZ4877 for EA provides compelling evidence that suggests that, within the confines of our study, values of *V*_T_ from tracer kinetics analysis can provide more robust evidence for assessing the efficacy of acupuncture. Also, clinical AD samples and human translation research on more specimens may need to be collected in the future to verify the preliminary animal study. We believe that if applied to clinical research, the precise acupuncture treatment protocols and quantitative assessment of AD could significantly promote nuclear medicine's role in AD therapies. In the future, our research group will actively promote the clinical translation of [^18^F]TZ4877 and strive to further verify our conclusions on patients with AD. Establishing this hypothesis will hopefully provide novel approaches to AD treatment. We believe that S1PR1 bridges acupuncture and CNS diseases providing a new perspective in both fields for clinical practice.

In conclusion, the function and mechanism of S1PR1 in the EA treatment of AD have not been previously documented. We initially confirmed that EA at “Baihui (GV20)‐Sishencong (EX‐HN1)” effectively improved cognitive function in AD mice by decreasing S1PR1 in the cortex and hippocampus. The co‐localization of S1PR1 with GFAP or IBA‐1 and the reduction of IL‐1β and TNF‐α suggested that EA may exert beneficial effects via an anti‐inflammatory effect as an alternative therapy for AD. Additionally, our study marked the first use of [^18^F]TZ4877 to assess the capacity of EA in the brains of AD mice. This study further promotes the exploratory application of PET quantifications for evaluating the efficacy of AD.

## CONFLICT OF INTEREST STATEMENT

All authors declare no financial interests or potential competing interests. A patent is pending regarding this new method for evaluation of the EA treatment (No. 202311832723.3). No other potential conflicts of interest relevant to this article exist. Author disclosures are available in the supporting information.

## ETHICS STATEMENT

All rodent study was conducted under the administration of the IACUC of the Guangdong Molecular Imaging Engineering Research Center in the Fifth Affiliated Hospital of Sun Yat‐sen University (ethical protocol number # 00274.1.2). Eight‐month‐old male APP/PS1 mice (C57BL/6) and age‐ and sex‐matched C57BL/6 mice were purchased from Cavens (Changzhou) Laboratory Animals, Ltd. (license No. SCXK [Su] 2021‐0013). All the animal experiments were performed under the National Institutes of Health Guidelines for the Care and Use of Laboratory Animals. The animals were housed in standard cages in the laboratory animal center of the Fifth Affiliated Hospital of Sun Yat‐sen University.

## Supporting information



Supporting Information

Supporting Information
