# Supplementary material for: Effectiveness of electro‐acupuncture for cognitive improvement on Alzheimer's disease quantified via PET imaging of sphingosine‐1‐phosphate receptor 1
Source: Alzheimers Dement. 2024 Sep 25;20(12):8331–45. doi: 10.1002/alz.14260 (PMC11667549; doi:10.1002/alz.14260)
Supplement: Supplementary file 1 — Supporting Information [file ALZ-20-8331-s002.docx]

**Supplementary Materials**

**Effectiveness of Electro-acupuncture for Cognitive Improvement on Alzheimer’s Disease Quantified via Positron Emission Tomography (PET) Imaging of Sphingosine-1-phosphate Receptor 1**

Lu Wang^1,2,3^, Lei Bi^2,3^, Yifan Qiu^2,3^, Guolong Huang^2,3^, Peizhen Ye^2,3^, Yongshan Liu^2,3^, An Li^1^, Xuan Yang^1^, Peining Shen^4^, Qi Zeng^1^, Hongyu Zhang^1^, Shengqiao Li^1, 2 *^, Hongjun Jin^2,3*^

1. Department of Chinese Medicine Oncology, Cancer Center, The Fifth Affiliated Hospital, Sun Yat-sen University, Zhuhai, Guangdong Province, 519000, China;
2. Guangdong Provincial Engineering Research Center of Molecular Imaging, The Fifth Affiliated Hospital, Sun Yat-sen University, Zhuhai, Guangdong Province, 519000, China;
3. Guangdong-Hong Kong-Macao University Joint Laboratory of Interventional Medicine, the Fifth Affiliated Hospital, Sun Yat-sen University, Zhuhai 519000, China
4. Pharmaceutical Clinical Trails Office, The Fifth Affiliated Hospital, Sun Yat-sen University, Zhuhai, Guangdong Province, 519000, China;

* Corresponding authors:

Shengqiao Li, M.D/ Ph.D., Department of Chinese Medicine Oncology, Cancer Center, The Fifth Affiliated Hospital, Sun Yat-sen University, Zhuhai, Guangdong Province, 519000, China; Email: [lishq26@mail.sysu.edu.cn](mailto:xxx@mail.sysu.edu.cn)

or

Hongjun Jin, Ph.D., Guangdong Provincial Engineering Research Center of Molecular Imaging, Guangdong-Hong Kong-Macao University Joint Laboratory of Interventional Medicine The Fifth Affiliated Hospital, Sun Yat-sen University, Zhuhai, Guangdong Province, 519000, China; Email: [jinhj3@mail.sysu.edu.cn](mailto:jinhj3@mail.sysu.edu.cn)

**Table S1. Summary of the primary apparatus**

| **Apparatus** | **Source** | **Details** |
| --- | --- | --- |
| Disposable sterile acupuncture needles | Suzhou Tianyi Acupuncture Equipment Co., LTD | Specifications: 0.16 × 7 mm |
| Electro-acupuncture apparatus | BeijingWarwick Industrial Development Company | Model: HANSLH202 |
| Morris Water Maze (MWM) | Morris water maze system (Shanghai Xinsoft Information Technology Co., Ltd., China) | Model: RWD MWM combined with Smart V3.0 camera system and Visu Track software |
| Image acquisition and analysis system | Visu Track Rodent Behavior Analysis Software (Shanghai Xinsoft Information Technology Co., Ltd., China) | Model: XR-VT |
| Magnetic Resonance Imaging | BioSpec 94/20USR, Bruker Inc., Germany | Echo Time = 22.50 ms, Repetition Time = 6200 ms, 5 averages, 5 of averages, 8 of RARE factor 8, slices with a thickness of 0.3 mm |
| Micro-positron emission  Tomography (PET) | NanoScan PET/CT 82S, Mediso Ltd., Hungary | Model: voxel size = 0.4 mm, Reconstruction approach was Ordered Subset Expectation Maximization, Random, attenuation, and scatter correction |
| PMOD Biomedical image quantification system | PMOD Technologies LLC, Switzerland | Model: For the analysis of mouse brain data the Mouse (Ma-Benveniste-Mirrione) VOI atlas is available |
| Laser scanning confocal microscope (LSCM) | Zeiss, Germany | Model: ZEISS LSM 880 |

**Table S2. Summary of regents and antibodies**

| **Agents** | **Source** | **Details** |
| --- | --- | --- |
| [^18^F]TZ4877 | Radio-synthesized by authors. | Radiochemical purity (> 95%), radiochemical yields (58.5 ± 12.7%), and specific activity (17.6 ± 5.3GBq/μmol) |
| [^18^F]AV45 | Guangdong Huixuan Inc Ltd, China | Radiochemical purity (91.60%), radiochemical yields (62%), and specific activity (0.389GBq/μmol) |
| Primary S1PR1 Antibody | Invitrogen, USA | Rabbit anti-S1PR1 polyclonal antibody, PA1-1040 |
| Primary GFAP Antibody | Abcam Inc., China | Chicken anti-GFAP polyclonal antibody, ab4674 |
| Primary  IBA-1 Antibody | Abcam Inc., China | Goat anti-Iba-1 polyclonal antibody, ab5076 |
| Secondary Antibody  (for S1PR1) | Invitrogen, USA | Goat Anti-Rabbit, 1937183 |
| Secondary Antibody  (for GFAP) | Jackson ImmunoResearch Inc., USA | Donkey Anti-Chicken, 34606ES60 |
| Secondary Antibody  (For IBA-1) | Jackson ImmunoResearch Inc., USA | Donkey Anti-Goat, 705-545-003 |
| DAPI | Thermo Fisher Scientific, USA | H-1200-10 |
| IL-1β | [Affinity Biosciences LTD](http://www.baidu.com/link?url=awFMr0F_BQp67R8-chEdHsIWpsmt38xx9C-jOd07BQSgePH2n-4kNfo7l1w5GFJG), China | Rabbit anti-IL-1β polyclonal antibody, AF5103 |
| TNF-α | [Affinity Biosciences LTD](http://www.baidu.com/link?url=awFMr0F_BQp67R8-chEdHsIWpsmt38xx9C-jOd07BQSgePH2n-4kNfo7l1w5GFJG), China | Rabbit anti-TNF-α polyclonal antibody, AF7014 |

**
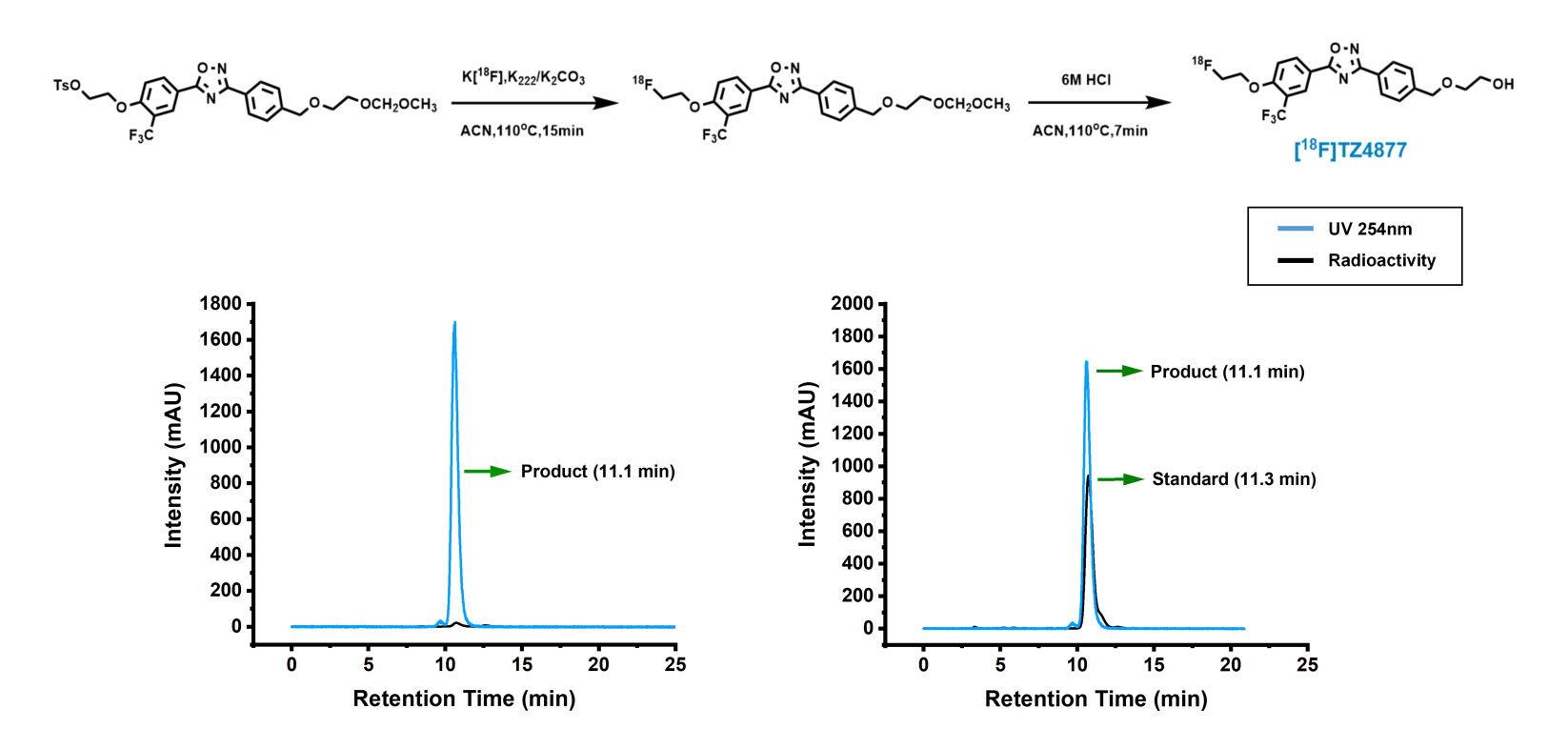
**

**Figure S1.** **Radiosynthesis and quality control for [^18^F]TZ4877.**


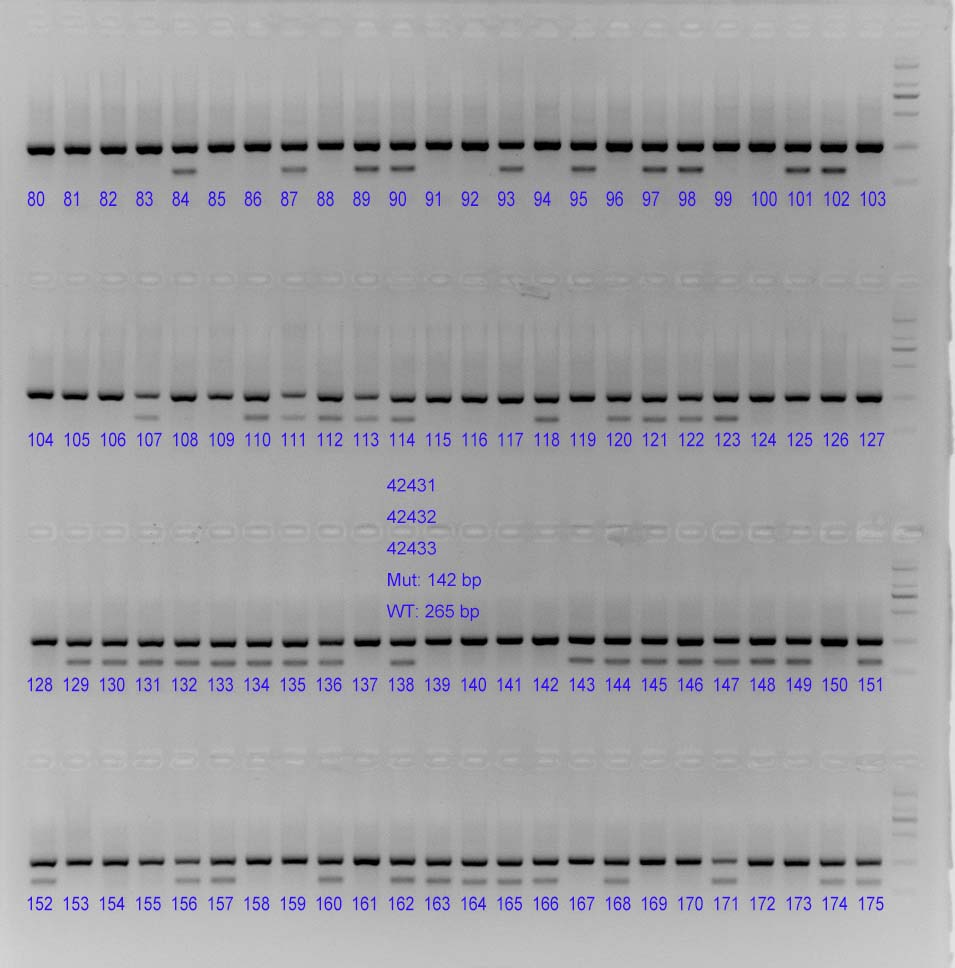


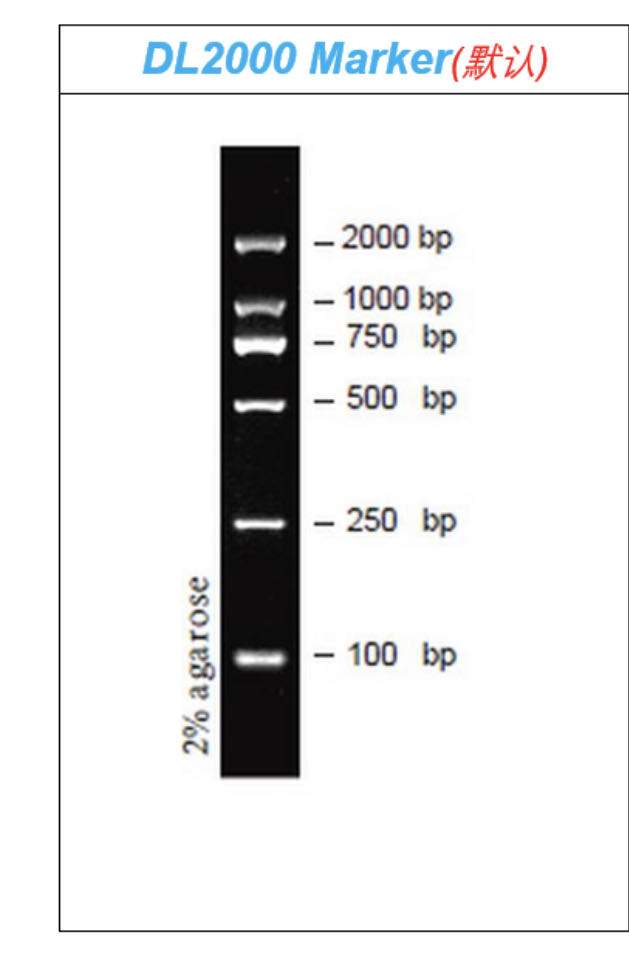

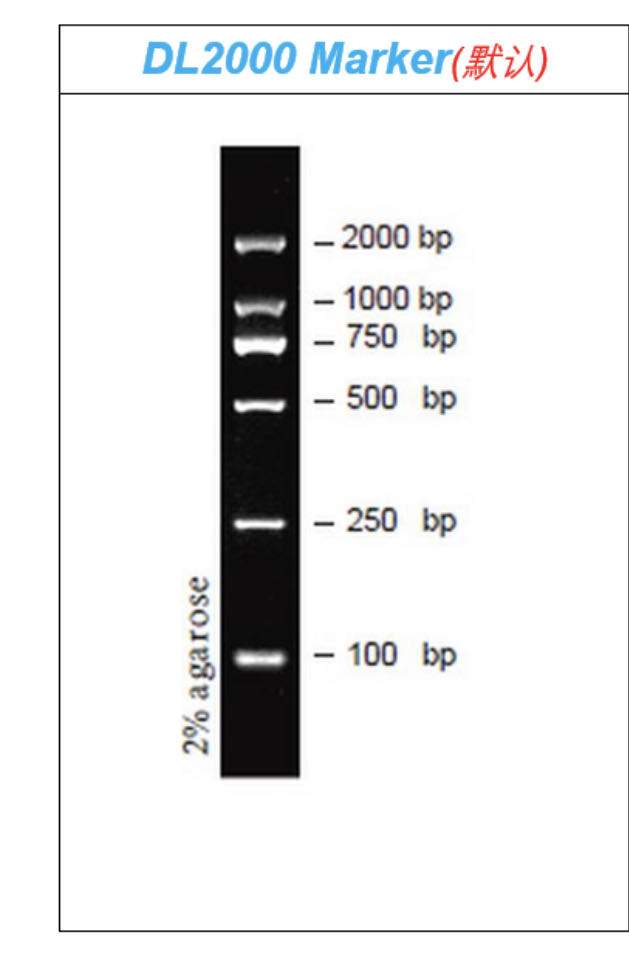

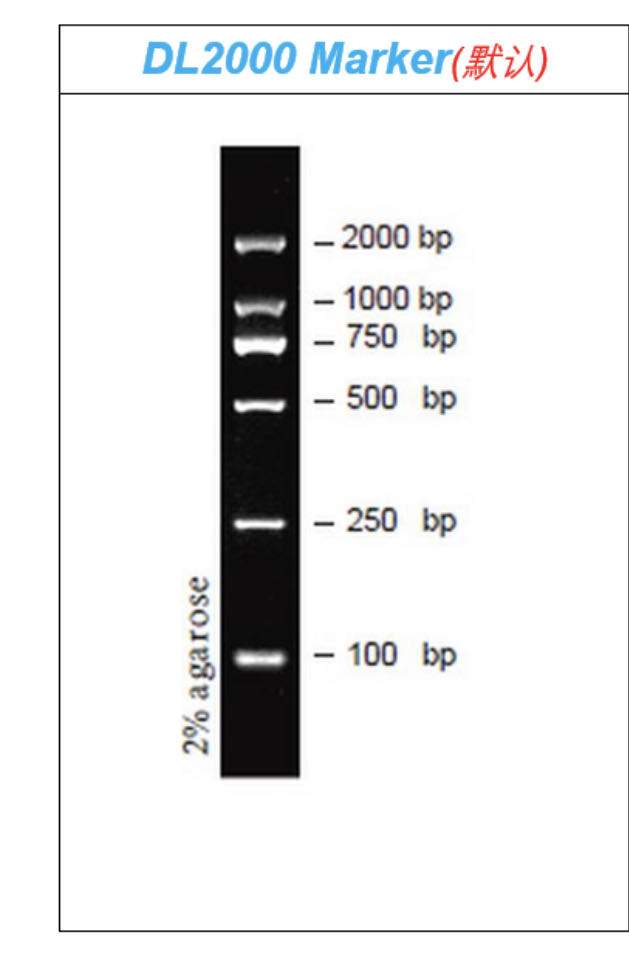

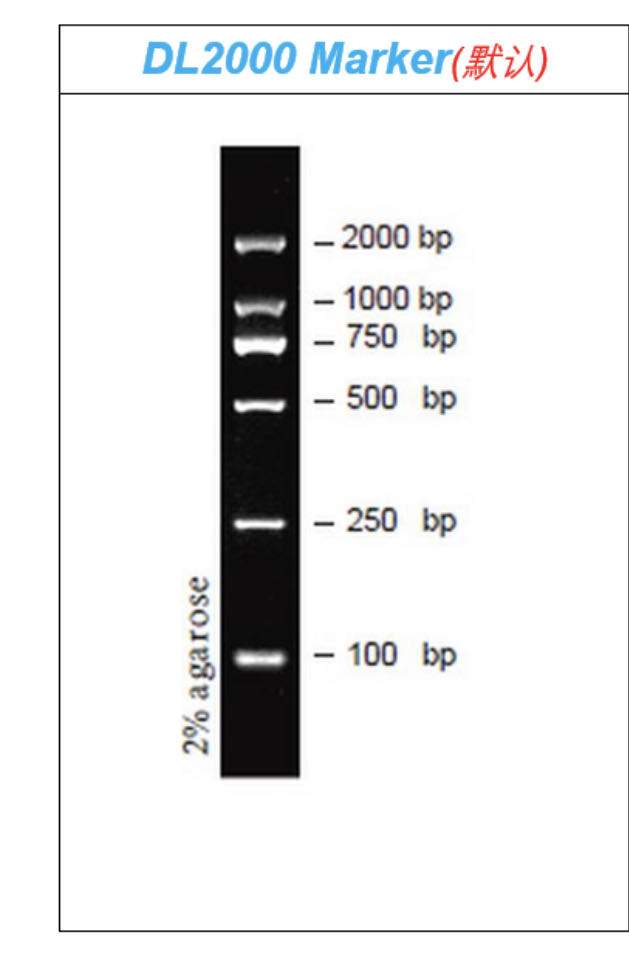


**Figure S2. The genetic test report of APP/PS1 transgenic mice.** The positive mice were identified with extra 142 bp bands under generic 265 bp bands. (Ten mice for this study are listed: #110; #111; #112; #113; #114; #118; #121; #123; #129; and #133)

**
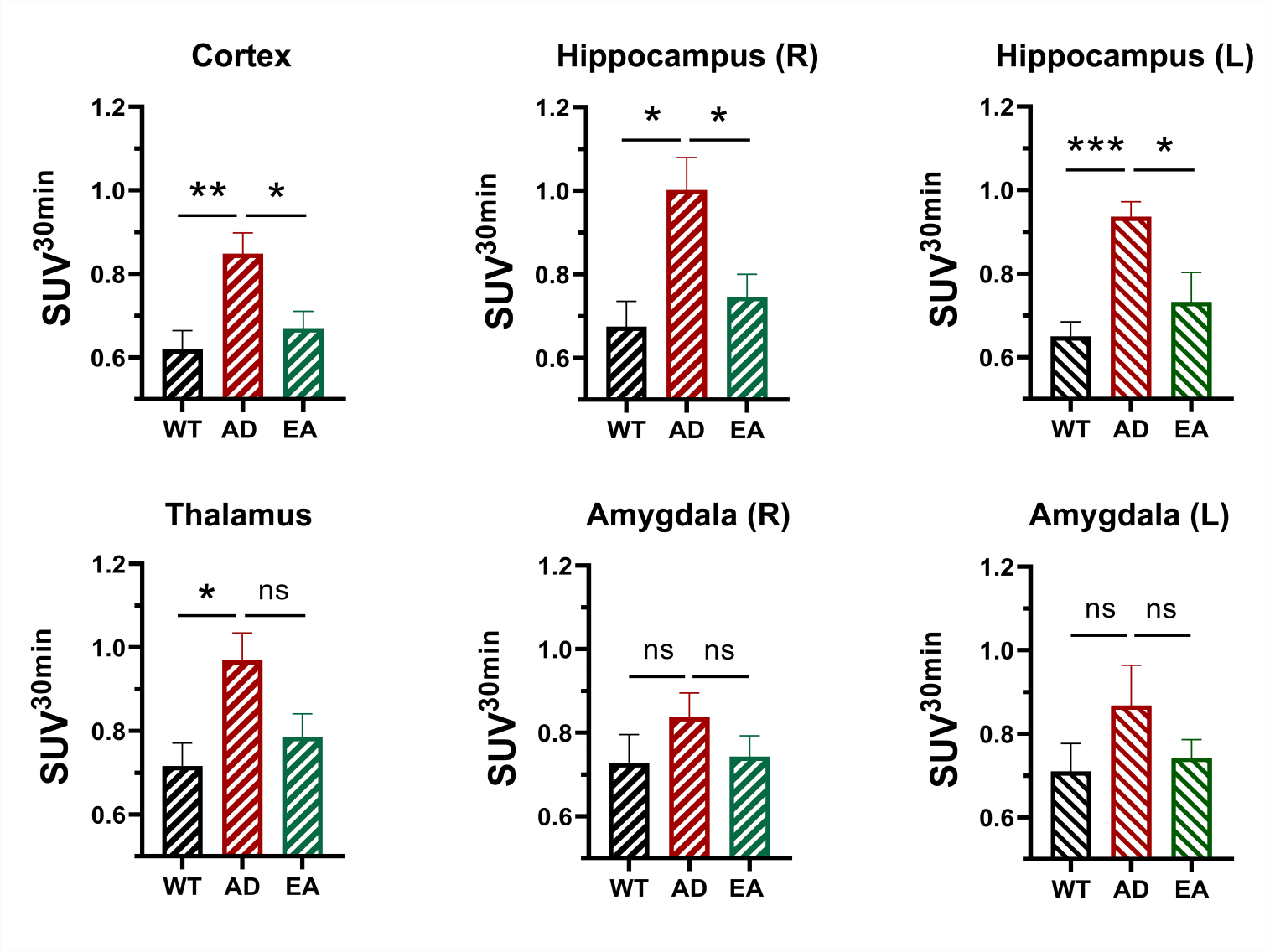
**

**Figure S3. The** **comparisons SUV of [^18^F]TZ4877** **(S1PR1) among WT, AD, and EA mice at 30 min post-injection.** Compared to the WT group (*n* = 5), a significant increase of [^18^F]TZ4877 uptake was found in AD mice (*n* = 5) at 30 min post-injection. Furthermore, the increased uptake in the AD brain can be visibly reduced especially in the cortex and hippocampus by EA (*n* = 5) treatment, rather than thalamus and amygdala. Although there was a slightly higher SUV in EA mice than in WT mice, the difference was not significant. (Key:* represents *p* < 0.05; ****** represents *p* < 0.01; ******* represents *p* < 0.001; ns represents *p >* 0.05.)


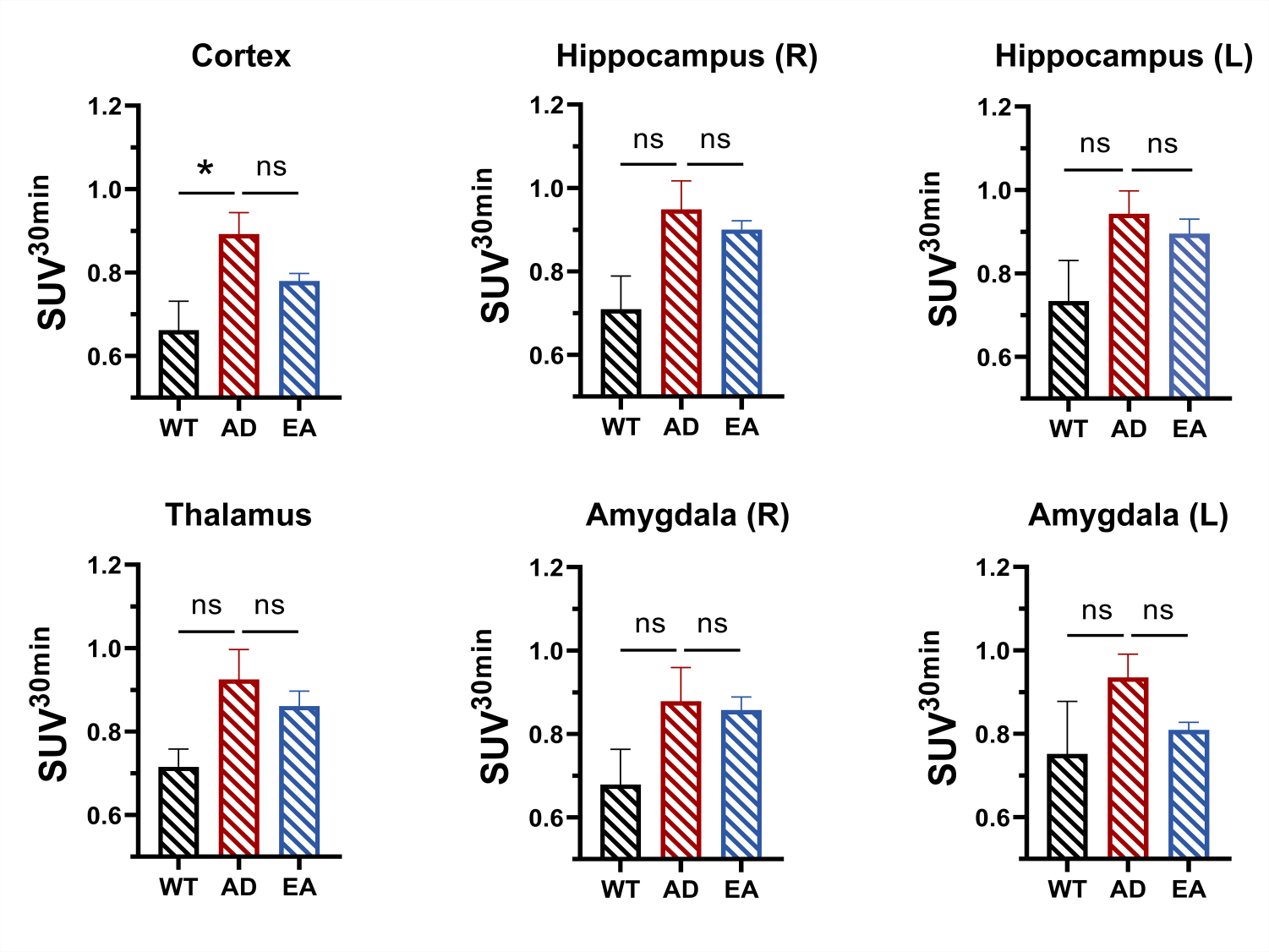


**Figure S4. The comparisons SUV of [^18^F]AV45 (Aβ) among WT, AD, and EA mice at 30 min post-injection.** To further investigate effectiveness of EA on AD, PET tracer [^18^F]AV45 was used in this study to detect Aβ deposition in mouse brain at 30 min post-injection. As shown in the Figure, an increased uptake of [^18^F]AV45 was found in AD mice (*n* = 5) compared to WT mice (*n* = 3), but only there was significance in the cortex and no statistical significance in the hippocampus. Similarly, EA treatment (*n* = 5) demonstrated a trend towards reduced [^18^F]AV45 uptake in the brains of AD mice post-treatment. However, there was no significant statistical difference in most brain regions, typical areas illustrated in the Figure. (Key: * represents *p* < 0.05; ns represents *p >* 0.05.)

**
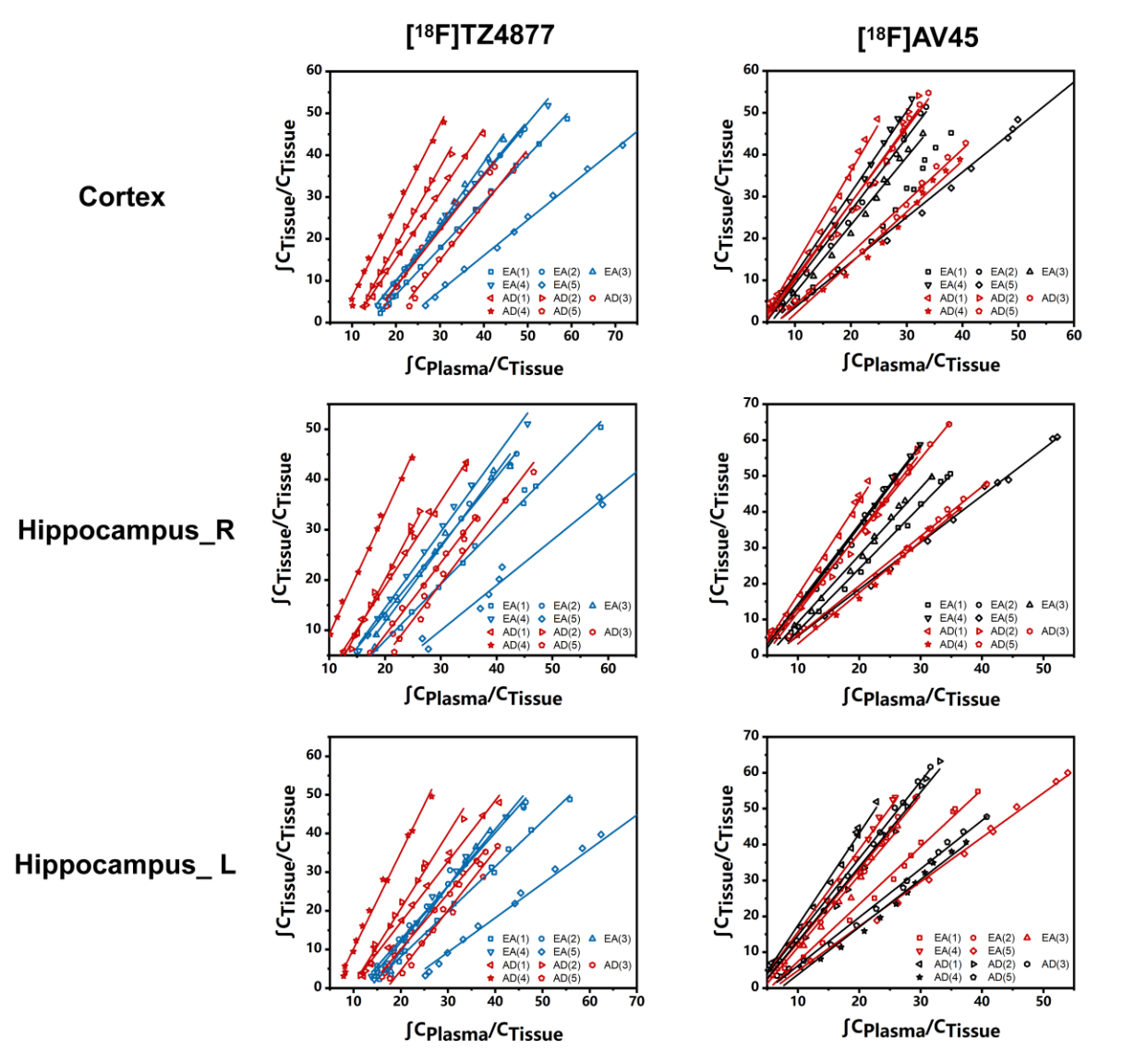
**

**Figure S5. The VT calculations Logan graphic analysis of [^18^F]TZ4877 (Left) and [^18^F]AV45 (Right) in AD and EA groups.** Degree of separation of oblique lines of Loan plots of [^18^F]TZ4877 or [^18^F]AV45 in certain regions of the brain in EA-treated and AD mice. Each line represents the 0-30 min dynamic PET quantifications from each mouse. For [^18^F]TZ4877 (Left), red lines represent AD mice and blue lines represent EA mice. For [^18^F]AV45 (Right), red lines represent AD mice and dark lines represent EA mice.


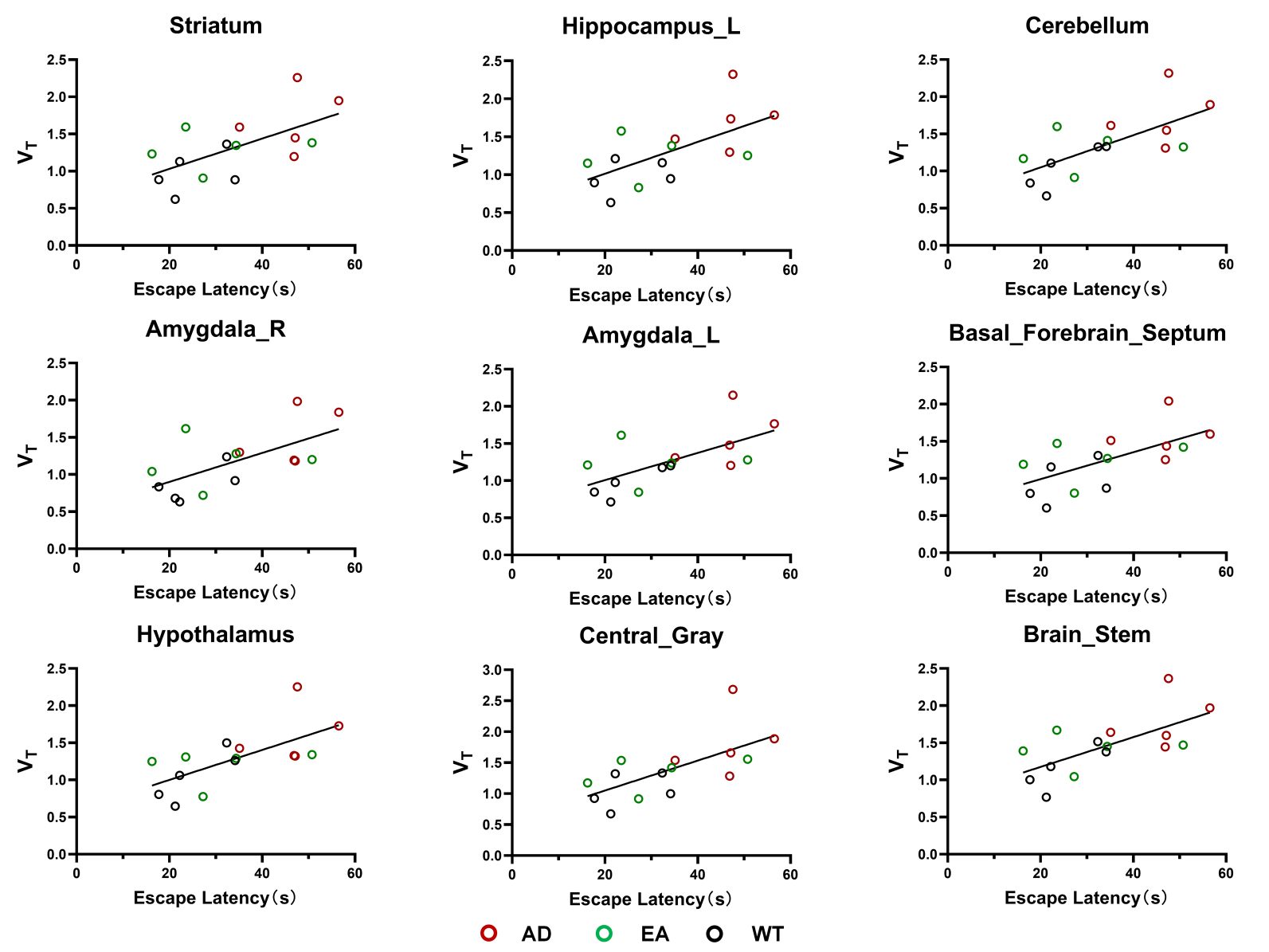


**Figure S6. The linear regression between V_T_ of [^18^F]TZ4877 (S1PR1) in brain areas and escape latency in hidden platform period** (Key: black circles for WT; green circles for EA, and red circles for AD).


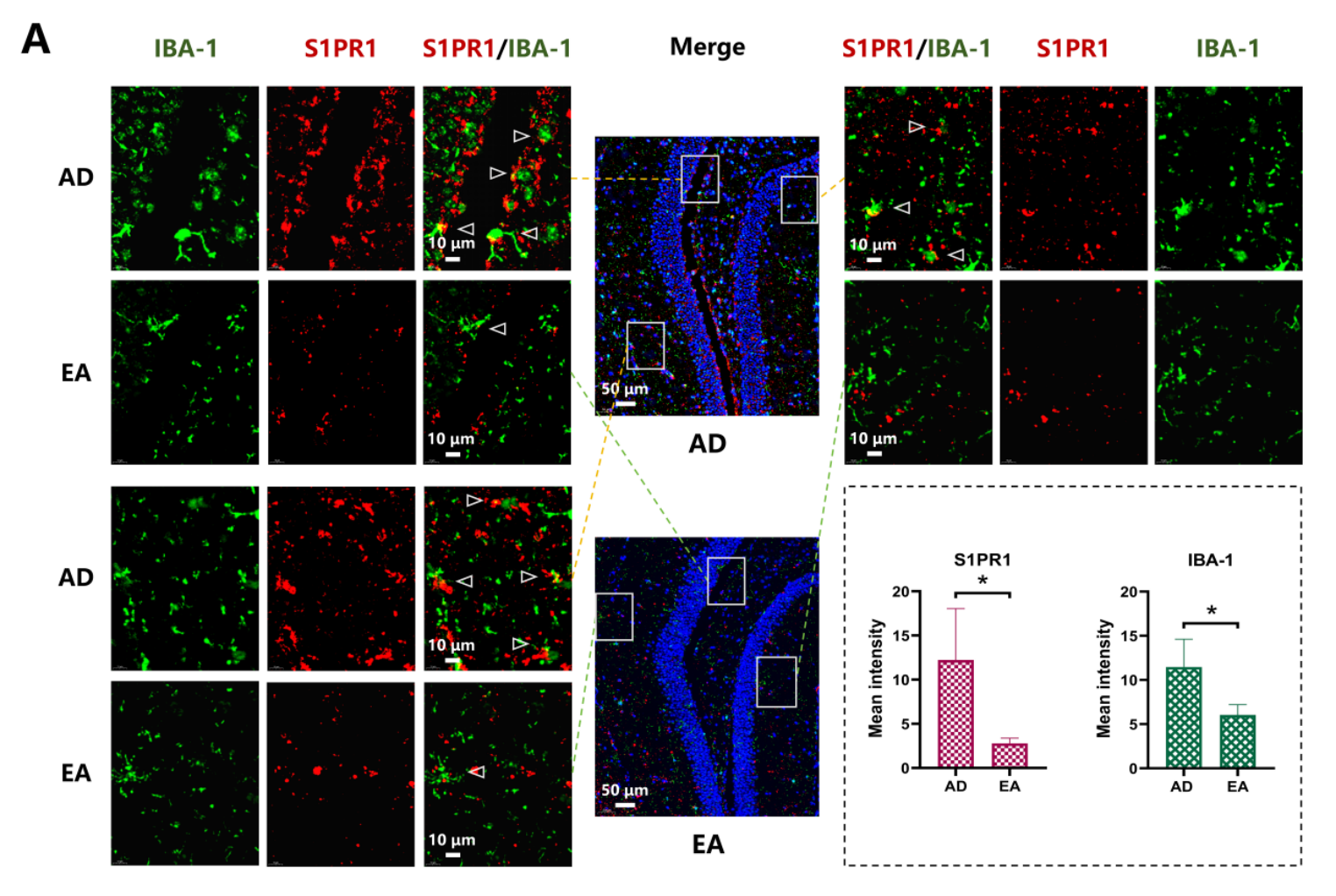


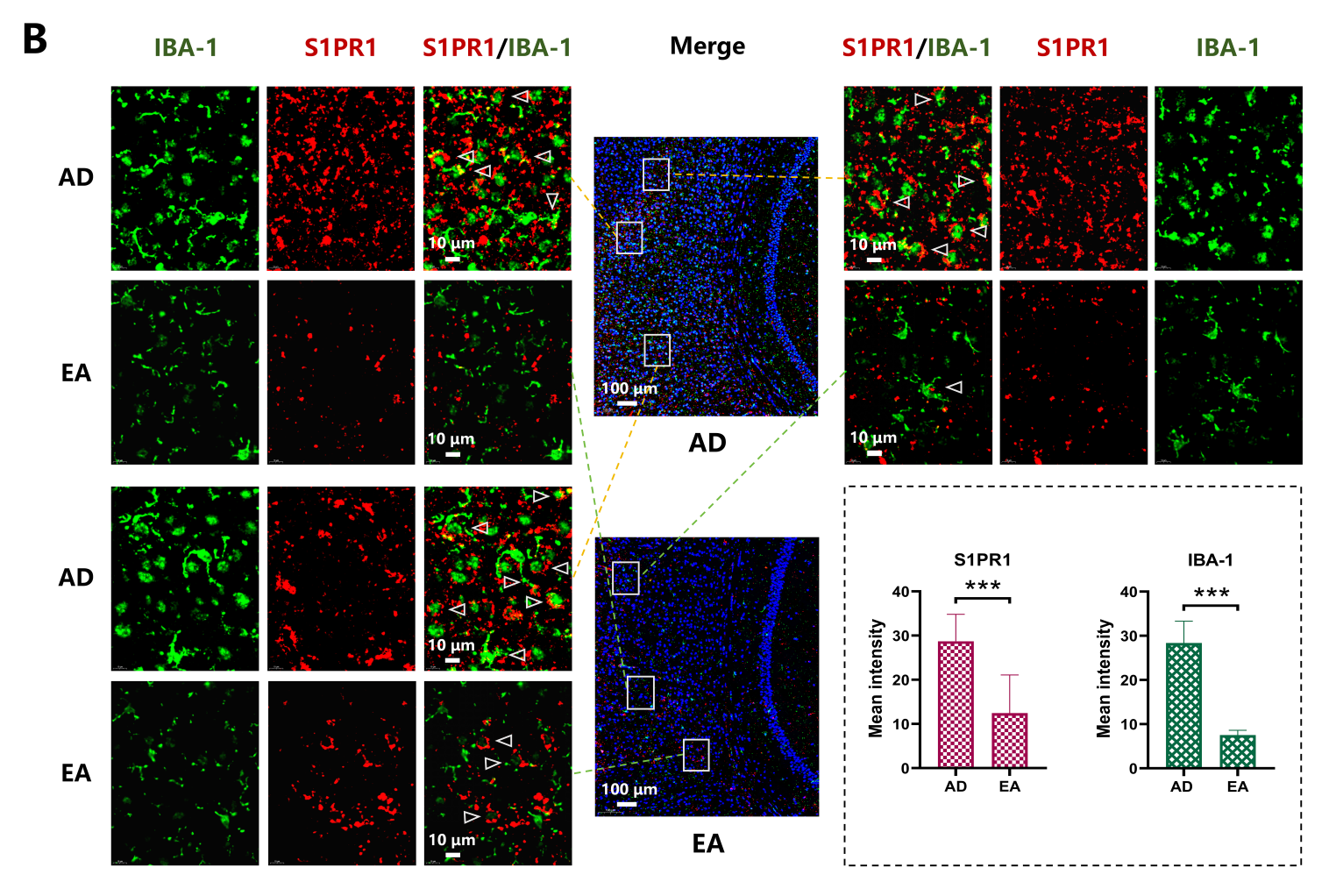


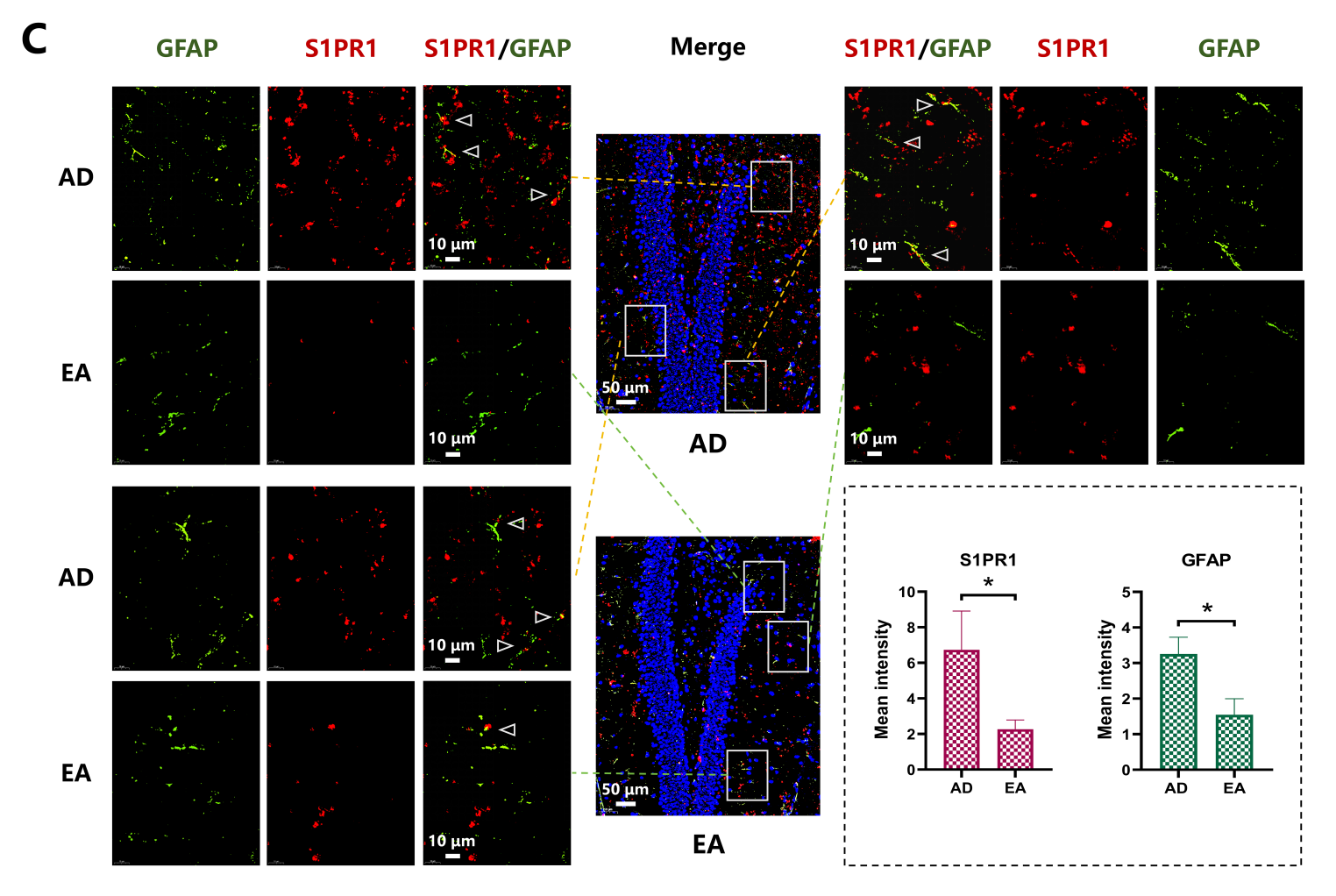


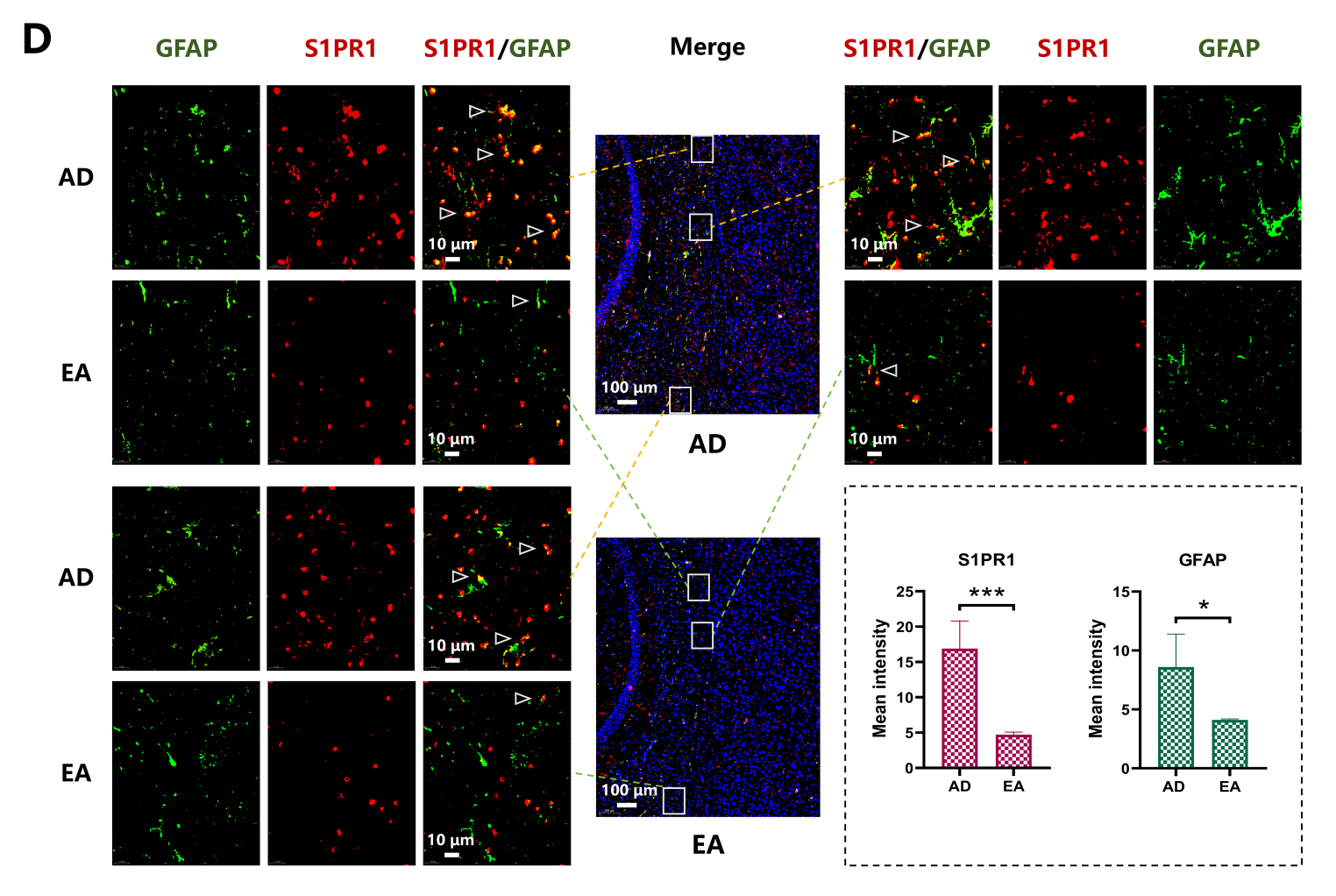


**Figure S7. The colocalization of S1PR1 with GFAP and IBA-1.** (A) Multiple immunofluorescence (IF) staining revealed colocalizations (white arrows) of S1PR1 (red), IBA-1 (green for microglia) and DAPI (blue) in hippocampus of AD and EA mice; (B) Multiple IF staining displayed colocalizations (white arrows) of S1PR1 (red), IBA-1 (green for microglia), and DAPI (blue) in cortex of AD and EA mice. (C) Multiple IF staining showed colocalizations (white arrows) of S1PR1 (red), GFAP (green for Astrocyte) and DAPI (blue) in hippocampus of AD and EA mice; (D) Colocalization (white arrows) of S1PR1 (red), GFAP (green for Astrocyte), and DAPI (blue) in cortex of AD and EA mice was also observed, along with quantitative results of the mean intensity in each figure (3 fields of view in each group). Additionally, EA treatment resulted in a significant reduction of the colocalization in figure A, B, C, and D. *: *p* < 0.05, **: *p* < 0.01.


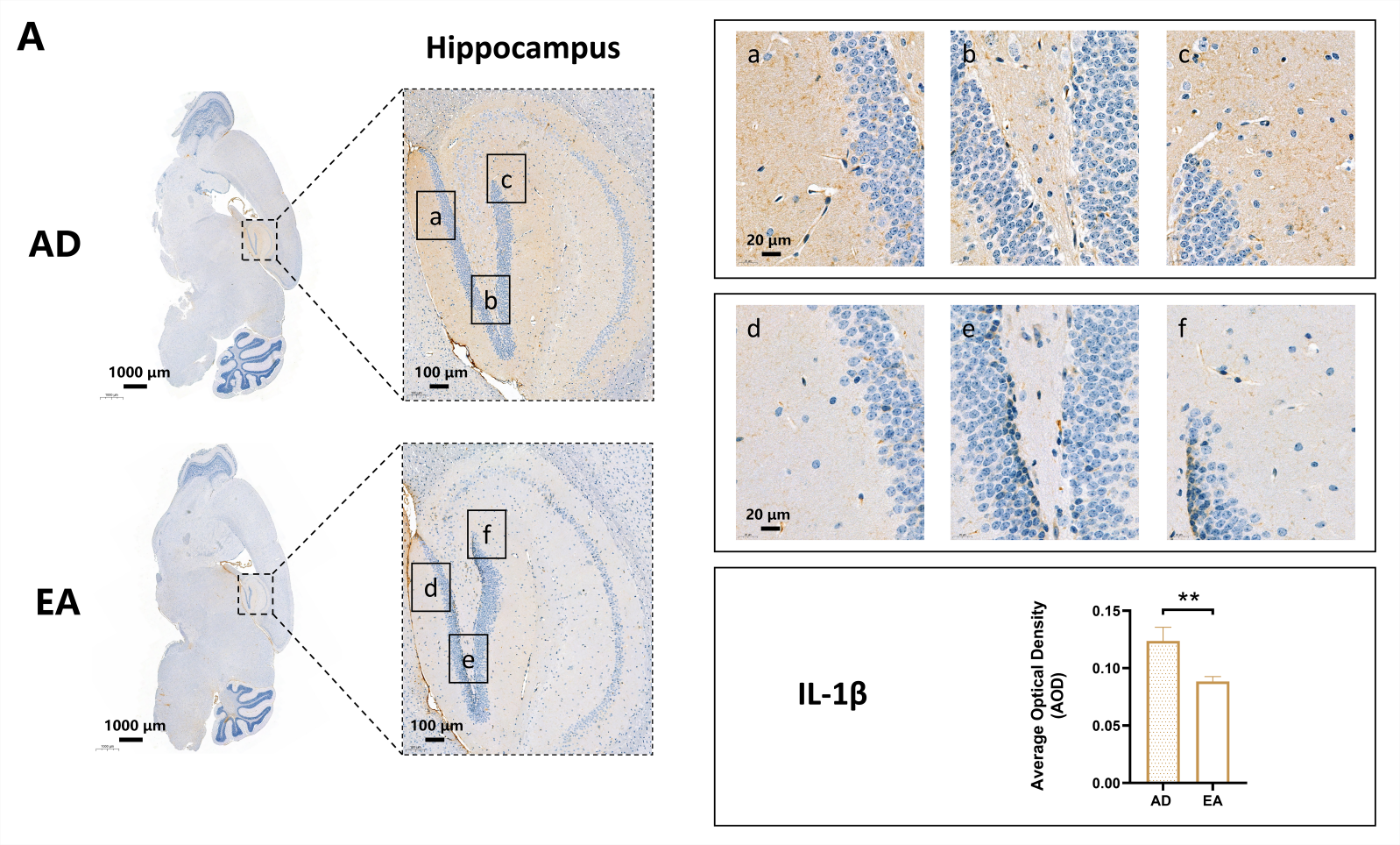


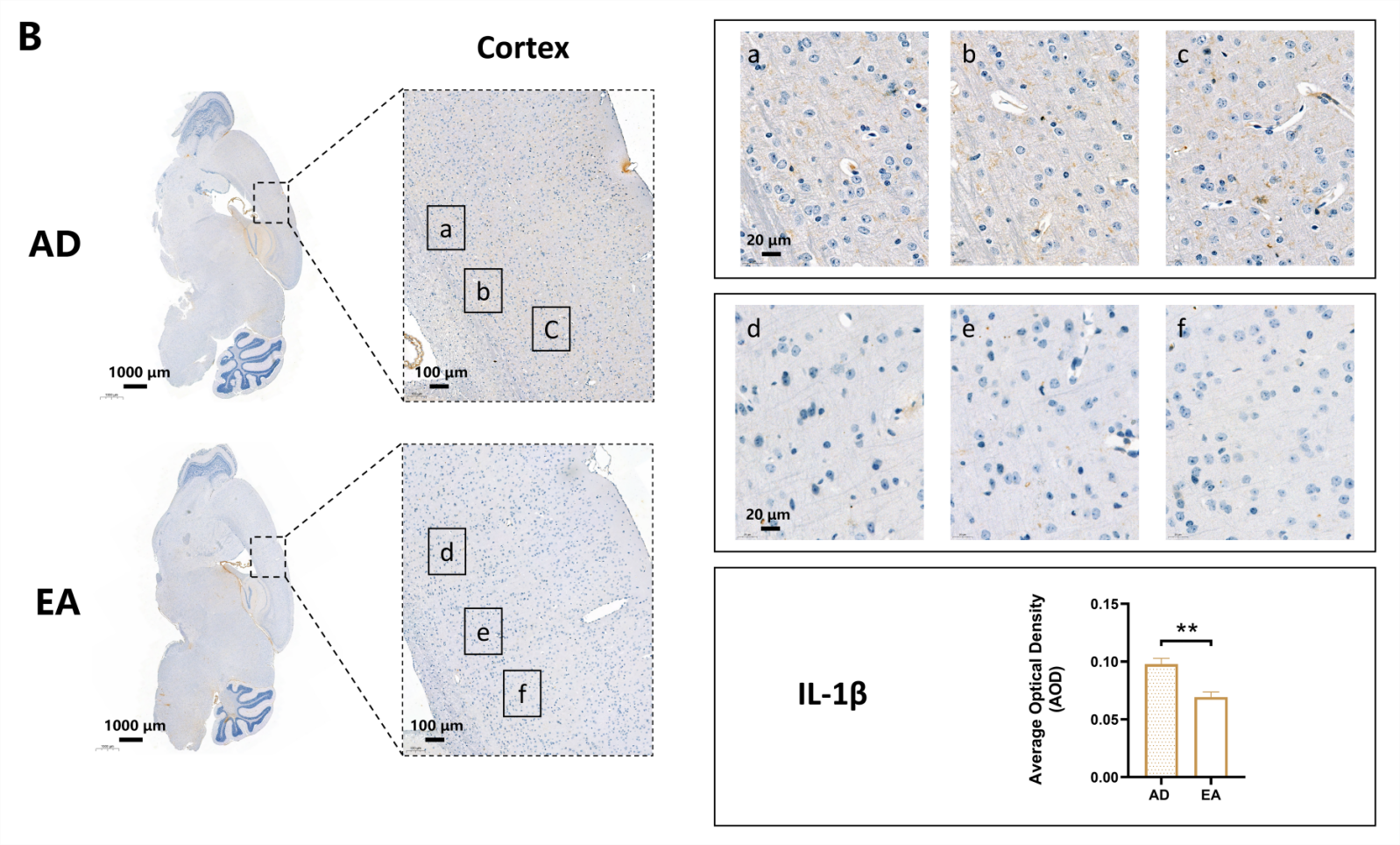


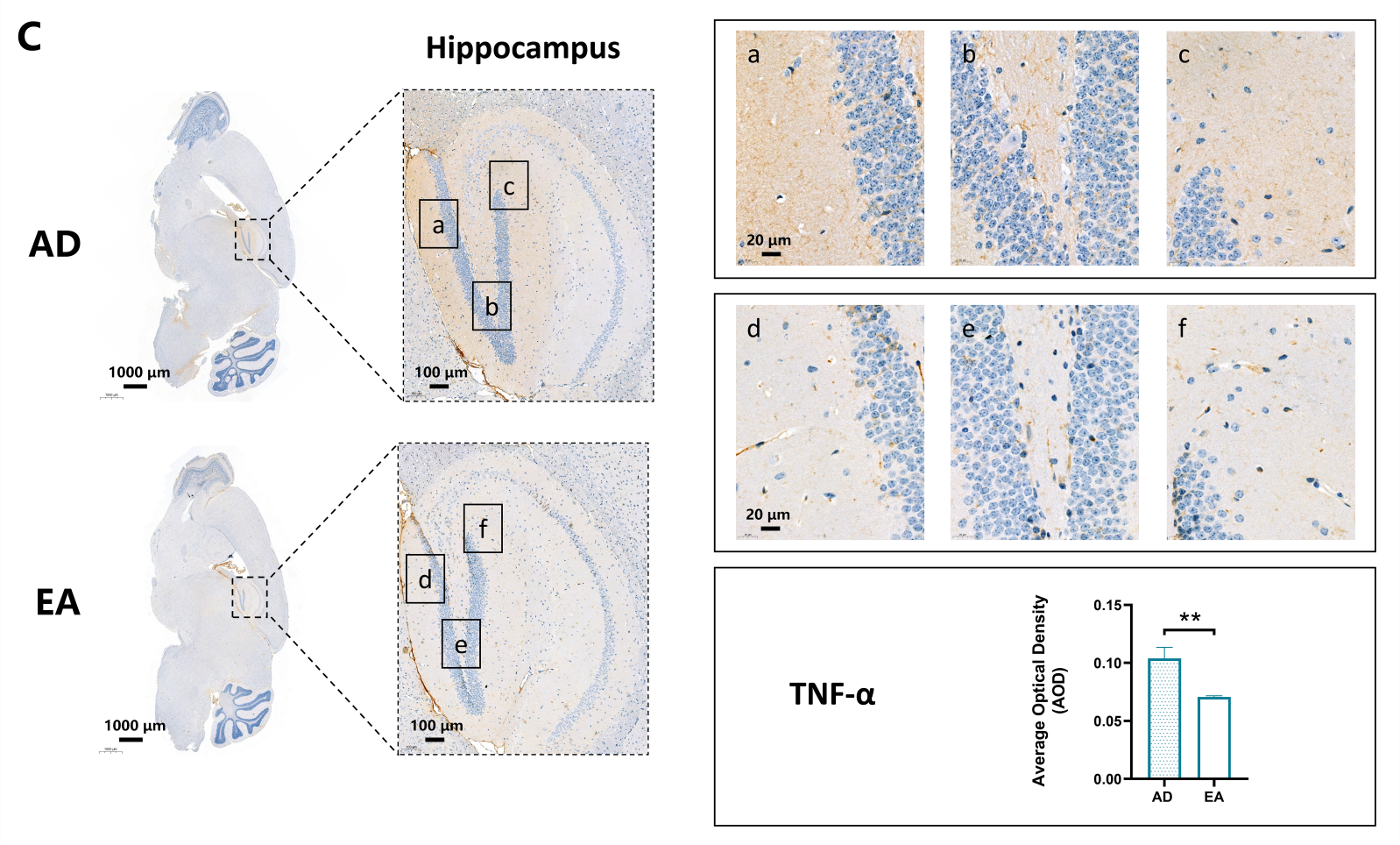


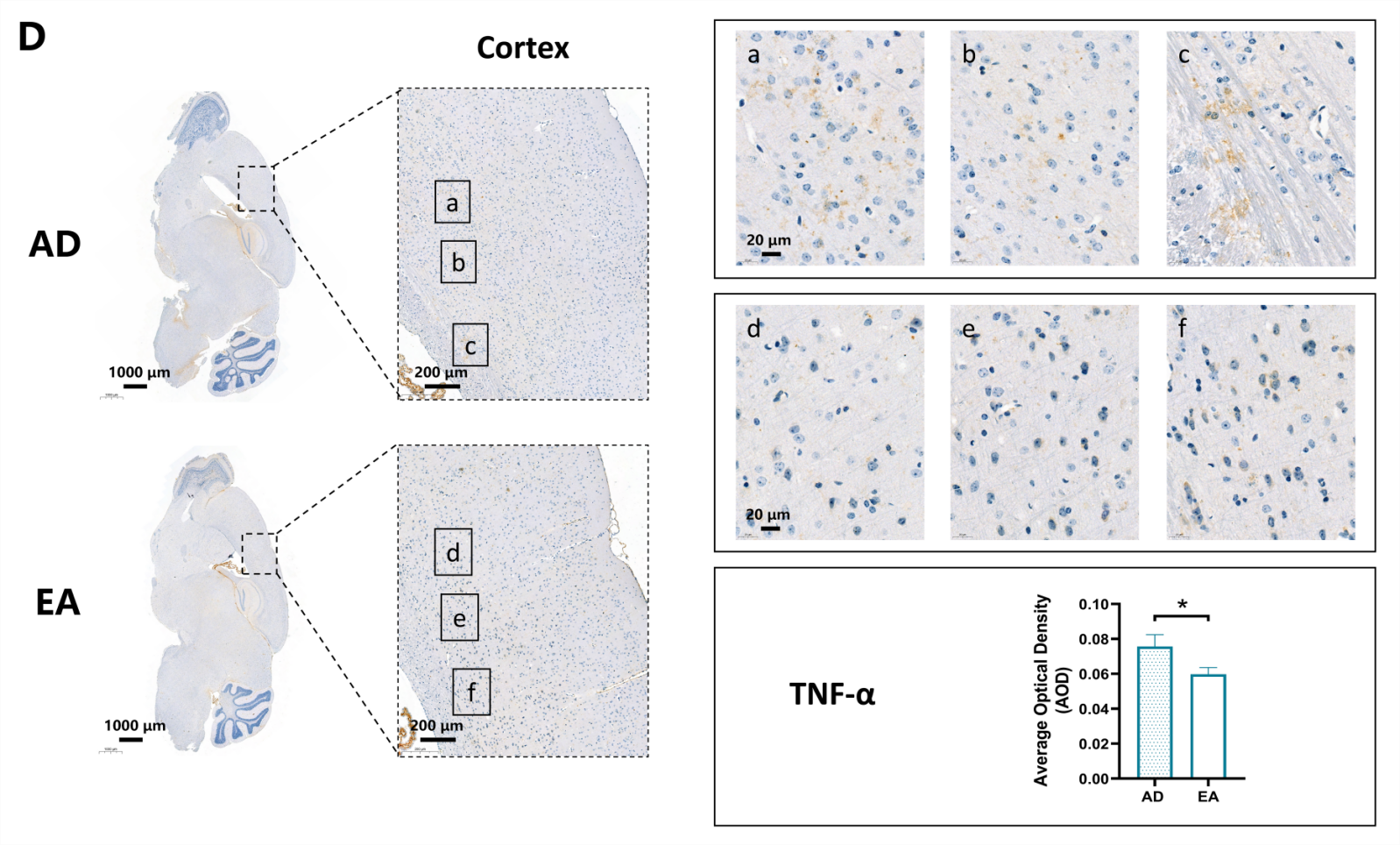


**Figure S8. Immunohistochemistry of IL-1β and TNF-α in the Hippocampus and Cortex.** (A) Expression of IL-1β in the hippocampus of AD and EA; (B) Expression of IL-1β in the cortex of AD and EA. (C) Expression of TNF-α in the hippocampus of AD and EA; (D) Expression of TNF-α in the cortex of AD and EA, along with quantitative results of the Average Optical Density (AOD) in each figure (3 fields of view in each group). Compared to AD mice, EA treatment resulted in a significant reduction of the expression of IL-1β and TNF-α in hippocampus and cortex. *: *p* < 0.05, **: *p* < 0.01.
